# Stromal architecture directs early dissemination in pancreatic ductal adenocarcinoma

**DOI:** 10.1172/jci.insight.150330

**Published:** 2022-02-08

**Authors:** Arja Ray, Mackenzie K. Callaway, Nelson J. Rodríguez-Merced, Alexandra L. Crampton, Marjorie Carlson, Kenneth B. Emme, Ethan A. Ensminger, Alexander A. Kinne, Jonathan H. Schrope, Haley R. Rasmussen, Hong Jiang, David G. DeNardo, David K. Wood, Paolo P. Provenzano

**Affiliations:** 1 Department of Biomedical Engineering, University of Minnesota, Minneapolis, Minnesota, USA.; 2 University of Minnesota Physical Sciences in Oncology Center, Minneapolis, Minnesota, USA.; 3 Department of Pathology and Immunology, Washington University School of Medicine, St. Louis, Missouri, USA.; 4 Masonic Cancer Center,; 5 Institute for Engineering in Medicine, and; 6 Stem Cell Institute, University of Minnesota, Minneapolis, Minnesota, USA.

**Keywords:** Cell Biology, Oncology, Cancer, Cell migration/adhesion, Diagnostic imaging

## Abstract

Pancreatic ductal adenocarcinoma (PDA) is an extremely metastatic and lethal disease. Here, in both murine and human PDA, we demonstrate that extracellular matrix architecture regulates cell extrusion and subsequent invasion from intact ductal structures through tumor-associated collagen signatures (TACS). This results in early dissemination from histologically premalignant lesions and continual invasion from well-differentiated disease, and it suggests TACS as a biomarker to aid in the pathologic assessment of early disease. Furthermore, we show that pancreatitis results in invasion-conducive architectures, thus priming the stroma prior to malignant disease. Analysis in potentially novel microfluidic-derived microtissues and in vivo demonstrates decreased extrusion and invasion following focal adhesion kinase (FAK) inhibition, consistent with decreased metastasis. Thus, data suggest that targeting FAK or strategies to reengineer and normalize tumor microenvironments may have roles not only in very early disease, but also for limiting continued dissemination from unresectable disease. Likewise, it may be beneficial to employ stroma-targeting strategies to resolve precursor diseases such as pancreatitis in order to remove stromal architectures that increase risk for early dissemination.

## Graphical abstract

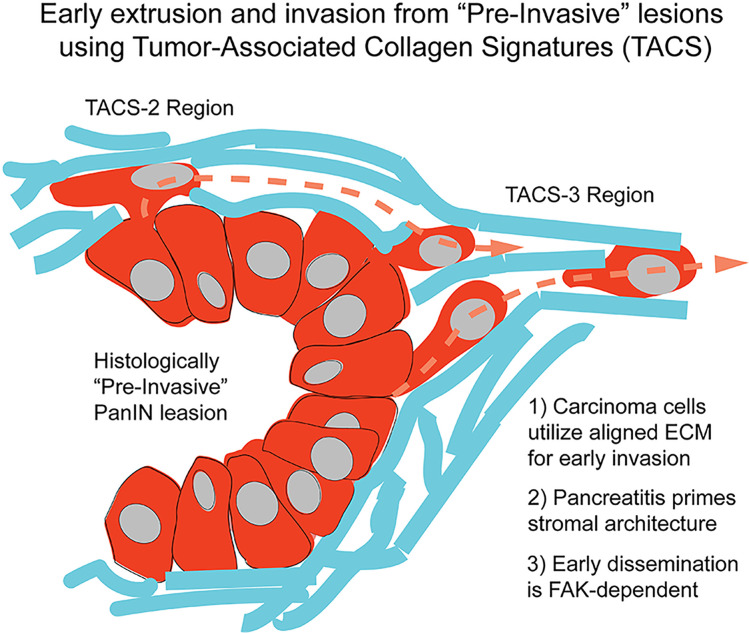

## Introduction

Pancreatic ductal adenocarcinoma (PDA) is a lethal disease with a dismal 5-year survival of ~9% (1). PDA is commonly characterized by a robust fibroinflammatory, or desmoplastic, response with hyperactivated stromal fibroblasts, robust immunosuppression, and vastly elevated extracellular matrix (ECM) deposition (2, 3). Apart from limiting the delivery and efficacy of chemotherapy (4–6) and immunotherapy (7, 8), the ECM of the desmoplastic stroma also plays a role in the extensive metastasis frequently observed in PDA (9, 10), and it may influence early dissemination. Indeed, recent studies using an autochthonous mouse model of PDA demonstrate that single carcinoma cells can disseminate into the ECM-rich stroma and peripheral blood even before frank histologically detectable malignancy (11). This is consistent with early disseminated cancer cells (DCCs) observed in breast cancer (12, 13) and decreased efficiency of dissemination in PDA following treatment with antifibrotic or antiinflammatory agents (7, 11), thus challenging the concept that metastasis is always a late event in cancer progression. Hence, the desmoplastic stroma appears to be a key player in early metastatic cell dissemination, yet the mechanisms by which the fibrotic stroma aids early dissemination are unknown.

One of the main components of the dense ECM in PDA is fibrillar collagen, which is thought to be deposited largely by activated pancreatic stellate cells in the tumor microenvironment (TME) (14, 15). Elevated collagen is a hallmark of several other desmoplastic solid tumors, including breast carcinoma, where it is associated with higher stiffness, hyperproliferation, increased invasion, and metastasis (16–19). In the context of invasion, both the amount of collagen and the architecture of the fibers in the TME are vitally important. In particular, several distinct collagen organization patterns, termed tumor-associated collagen signatures (TACS), have been identified in breast tumors with important implications in disease progression (20, 21). Among them were TACS-2 and TACS-3, both comprised of organized collagen fibers in the periductal space. For TACS-2, collagen fibers of variable density are mainly organized approximately parallel to either the ductal or carcinoma in situ boundary, around carcinoma cell clusters within the tumor mass, or at the tumor boundary. For TACS-3, the fibers orient perpendicularly to the cell clusters or ductal boundary, or they result in aligned collagen regions throughout the tumor mass in later stages, often providing a conduit for carcinoma cell invasion (20, 22). Consequently, due to contact guidance where cells utilize anisotropy from aligned ECM fibers to orient and migrate along single fibers, TACS-3–like aligned collagen patterns lead to increased focal and local invasion and metastasis (20, 22) and correlate with worse survival in patients (21). However, while recent studies suggest the presence of TACS architectures in PDA (10, 23), the prevalence of TACS in PDA, particularly relative to disease stage and early dissemination, remains largely unexplored. Thus, we hypothesized that TACS or TACS-like collagen architectures are involved in early dissemination and invasion in PDA.

Here, we employed an autochthonous mouse model of PDA, highly faithful to the human disease (24) and expressing carcinoma cell–specific fluorophores (10), as well as human PDA samples to define a key link between early dissemination in PDA and periductal collagen organization. We utilized integrated multiphoton excitation (MPE) and second harmonic generation (SHG) imaging on archival and live tumor samples to characterize the prevalence of TACS in PDA and demonstrate that periductal collagen patterns drive carcinoma cell dissemination. We further identified these patterns in pancreatitis and inflamed “normal” adjacent tissue, suggesting that precancerous fibroinflammatory disease can precondition and prime the stroma for early dissemination prior to transformation. Moreover, we identified focal adhesion kinase (FAK), a key mechanotransduction kinase molecule, as a regulator of the processes of ECM-guided extrusion from ductal epithelium and subsequent invasion, and we observed that blocking FAK function reduces the efficiency of single-cell dissemination and metastasis in PDA.

## Results

### Evolution of collagen organization in PDA.

**Figure 1:**
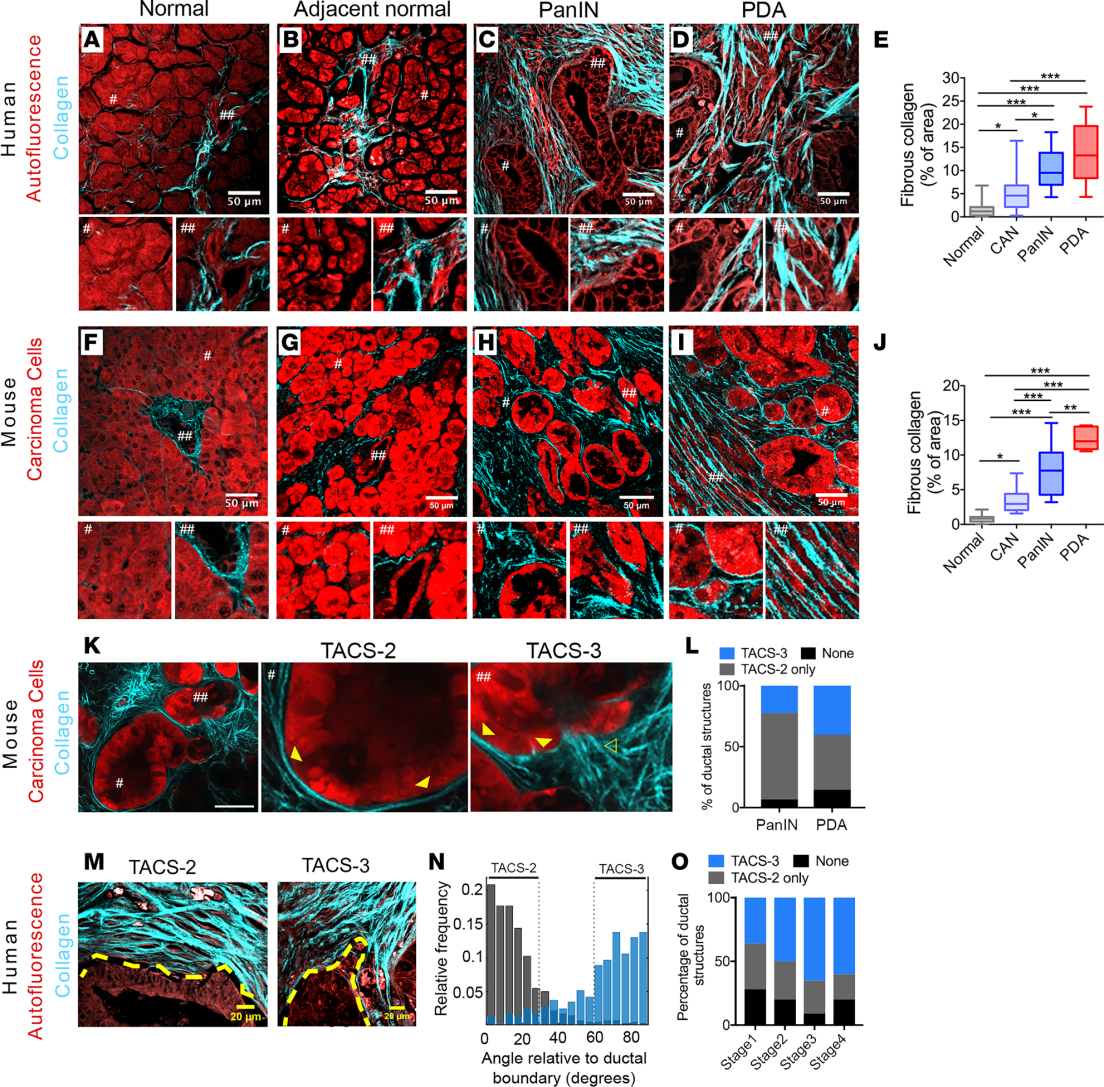
Fibrous collagen architectures in PDA. (**A**–**D**) Combined MPE and SHG imaging of patient samples showing tissue architecture by autofluorescence and fibrous collagen by SHG in (**A**) normal, (**B**) cancer-adjacent normal, (**C**) PanIN, and (**D**) mature PDA (# and ##, 2× magnifications of the indicated regions) and related (**E**) collagen quantification (*n* = 10–21 fields of view [FOV] across *n* ≥ 3 patient samples per group). (**F**–**I**) Combined MPE/SHG imaging and analysis using *KPCT* or *KPCG* mouse models of PDA for (**F**) normal, (**G**) cancer-adjacent normal, (**H**) PanIN, and (**I**) well-differentiated PDA and related (**J**) collagen quantification (*n* = 5–12 FOV across *n* ≥ 3 mice per group). (**K**) Typical collagen architectures in early disease (1.5-month *KPCT* mouse) (# and ##, 3× magnifications of the indicated regions; outlined and solid arrowhead, TACS-3 and TACS-2, respectively). (**L**) Frequency of quantified TACS (per duct) associated with PanIN and well-differentiated PDA in *KPCT/KPCG* mice (*n* > 20 FOV across ≥ 6 mouse tumors/group; by 2-way ANOVA, *P* < 0.0001 for TACS type and ns for PanIN/PDA as main effects; ns for association of each of None, TACS-2 only, and TACS-3 with PanIN versus PDA by Tukey’s multiple-comparison test). (**M** and **N**) Representative TACS-2^+^ and TACS-3^+^ ducts in well-differentiated human PDA, with yellow dashed lines indicating ductal boundaries (**M**), quantified by CT-FIRE (60) (**N**). (**O**) Frequency of TACS-2 and TACS-3 associated with ductal structures from different stages of human disease (*n* = 10–33 FOV across *n* ≥ 3 patient samples; *P* = 0.15 and *P* = 0.41 by Fisher’s exact test for the association of stage with None versus TACS and TACS-2 only versus TACS-3, respectively). Scale bars: 50 μm (**A**–**D**, **F**–**I**, and **K**) and 20 μm (**M**); box-and-whisker plots show minimum to maximum with median and interquartile range for **E** and **J**; *P < 0.05, **P < 0.01, ***P < 0.001 by the nonparametric Kruskal-Wallis test and Dunn’s multiple-comparison test.

To characterize the deposition and architecture of fibrillar collagen with disease progression in PDA, we utilized SHG imaging for label-free detection of collagen from live and archival murine and human tissue samples (Figure 1). Multiple large (~1 mm^2^), patient samples on a tissue microarray (complete with tumor staging and grading information) were imaged at high resolution by multiphoton microscopy to generate simultaneous SHG and MPE of endogenous cellular fluorescence (Supplemental Figure 1A; supplemental material available online with this article; https://doi.org/10.1172/jci.insight.150330DS1), enabling quantification of fibrous collagen from the SHG signal (Supplemental Figure 1B), while also mapping the localization of the collagen fibers with respect to tissue architecture. In normal pancreata, little collagen was observed, largely concentrated around the few ducts interspersed in tissue otherwise dominated by acinar cells (Figure 1, A and E; note no to low collagen surrounds acinar cells; see magnified region #) in contrast to robust collagen surrounding ductal structures (see magnified region ##). Notably, cancer-adjacent normal (CAN) regions showed elevated levels of collagen around ductal structures and in some regions between acinar clusters (Figure 1, B and E; note acinar regions can possess both collagen [main image] or no collagen regions [see magnified region #]), suggesting regional activation of a fibroinflammatory stromal response in CAN tissue, consistent with the link between fibroblast activity and collagen deposition in PDA development (3, 25). This may contribute to epithelial dysfunction in adjacent regions (e.g., acinar dropout, ductal hyperplasia, or acinar-to-ductal metaplasia [ADM]) and/or prime adjacent regions for invasion. Moreover, consistent with the desmoplastic response associated with PDA, as expected, robust fibrous collagen was ubiquitous in the periductal areas around pancreatic intraepithelial neoplasia (PanIN) lesions, which are histologically well-defined precursors to invasive ductal pancreatic adenocarcinoma (Figure 1, C and E). Elevated collagen remained pervasive throughout disease progression to mature PDA (Figure 1, D and E).

To complement analyses from patient samples, we employed the genetically engineered *Kras^LSLG12D/+^;p53^LSL-R172H/+^;Pdx-1-Cre* (*KPC*) mouse model that faithfully replicates the human disease, including the stromal response associated with PDA (5, 24), and provides an excellent tool to examine collagen organization during PDA progression. Here, we utilized fluorescent reporter *KPC* mice, namely *KPC;ROSA26^LSL-tdTomato/+^* (*KPCT*) or *KPC;ROSA26^LSL-ZsGreen1/+^*(*KPCG*; pseudo-colored red throughout figures for consistency unless otherwise noted) and performed similar analyses of the localization and distribution of collagen (Figure 1, F–J). Consistent with the human data, normal murine tissues possessed minimal stromal collagen while CAN regions showed regions of elevated collagen deposition around ducts that extend into the periacinar space (Figure 1, G and J). Likewise, histologically “preinvasive” PanIN regions were highly collagenous, with robust periductal collagen observed around the majority (>95%) of the ductal structures (Figure 1, H and J). Overall, these data from human and murine PDA samples demonstrate biased collagen localization around ductal structures in normal pancreata, elevated collagen levels in adjacent normal regions, and more robust collagen in early PanIN lesions and PDA.

Since elevated collagen levels in breast carcinomas are organized into specific architectures like TACS-3 — which guide local invasion (16, 20, 22) (Supplemental Figure 1C) — and both TACS-3 architectures (5, 10, 23) and early invasion (11) have been observed in PDA, we hypothesized that such architectures may also be prevalent in very early (histologically preinvasive) pancreatic cancer and play key roles in early invasion. This prompted us to characterize the periductal collagen organization in histologically early disease from *KPC* mice. Analysis of collagen architecture in the periductal space demonstrates that ducts are TACS-2^+^ (Figure 1, F–I). That is, analysis of SHG images shows parallel collagen distributed around approximately 0° (defined as ± 30°, similar to our previous reports; refs. 20, 26) relative to the epithelial boundary (Supplemental Figure 1C; see also magnified regions ## of ducts in Figure 1, F and G; TACS-2 organization shown in magnified regions # of PanINs; and well-differentiated PDA regions in Figures 1, H and I). We note that, in pancreatic disease, collagen is often less tightly bound around ductal structures than we previously observed when defining TACS-2 architectures found in mammary carcinomas (20). Furthermore, analysis of TACS-2^+^ ducts from early disease in approximately 1.5-month-old mice (preceding frank tumor formation) indicates that many of the ducts are also TACS-3^+^ (defined as collagen aligned perpendicular, approximately 90° ± 30°, similar to our previous reports, relative to the epithelial boundary; refs. 20, 26) (Supplemental Figure 1, C–E). That is, regions of the duct are positive for TACS-2, while adjacent regions of the ducts are positive for TACS-3 (Figure 1K; see also TACS-2 and TACS-3 architectures surrounding PanIN lesions in Figure 1H and Supplemental Figures 1E), demonstrating that collagen surrounding preinvasive PanIN lesions possess architectures that are known to promote invasion of pancreatic carcinoma cells (10). We note that both TACS-2 and TACS-3 architectures also exist in well-differentiated PDA regions (i.e., right half of Figure 1I), demonstrating that such aligned collagen architectures are maintained near ductal structures associated with more advanced disease. Strikingly, a significant fraction of the ductal structures in early disease present with TACS-3 regions, and indeed, the frequencies of individual TACS or their absence were not significantly different between early and mature disease (Figure 1L).

In agreement with murine PDA data, both TACS-2 and -3 are found surrounding ductal structures in human pancreatic cancer. TACS-2 is again robustly observed around the vast majority of ductal structures in early and well-differentiated PDA regions (Figure 1, C and D). Frequently, TACS-3 is observed locally, often associated with invaginations and regions of irregular ductal boundaries (Figure 1, M–O). Indeed, similar to findings in *KPC* mice, quantitative analysis of fiber orientations using a curvelet transform of the SHG signal demonstrates TACS-2 or TACS-3 periductal collagen organization (Figure 1N). Notably, in human samples with stage I to stage IV disease, the presence of TACS architectures was not limited to a particular disease stage, with no significant association between stage and either the presence of TACS or its particular type (Figure 1O). Indeed, a substantial proportion (~40%) of ductal structures in stage I patients also present with TACS-3 (Figure 1M), demonstrating that these conduits for invasion are present at early stages and remain prevalent in more advanced PDA. Thus, our findings that robust TACS-3 surrounding ductal structures emerges with histologically preinvasive disease and is maintained through advanced well-differentiated disease, combined with our understanding of the established role of aligned collagen in promoting highly directed carcinoma cell invasion (10, 22, 27–31), motivated us to further evaluate the influence of collagen alignment on early dissemination of pancreatic carcinoma cells.

### Single-cell dissemination along local periductal collagen architectures.

**Figure 2:**
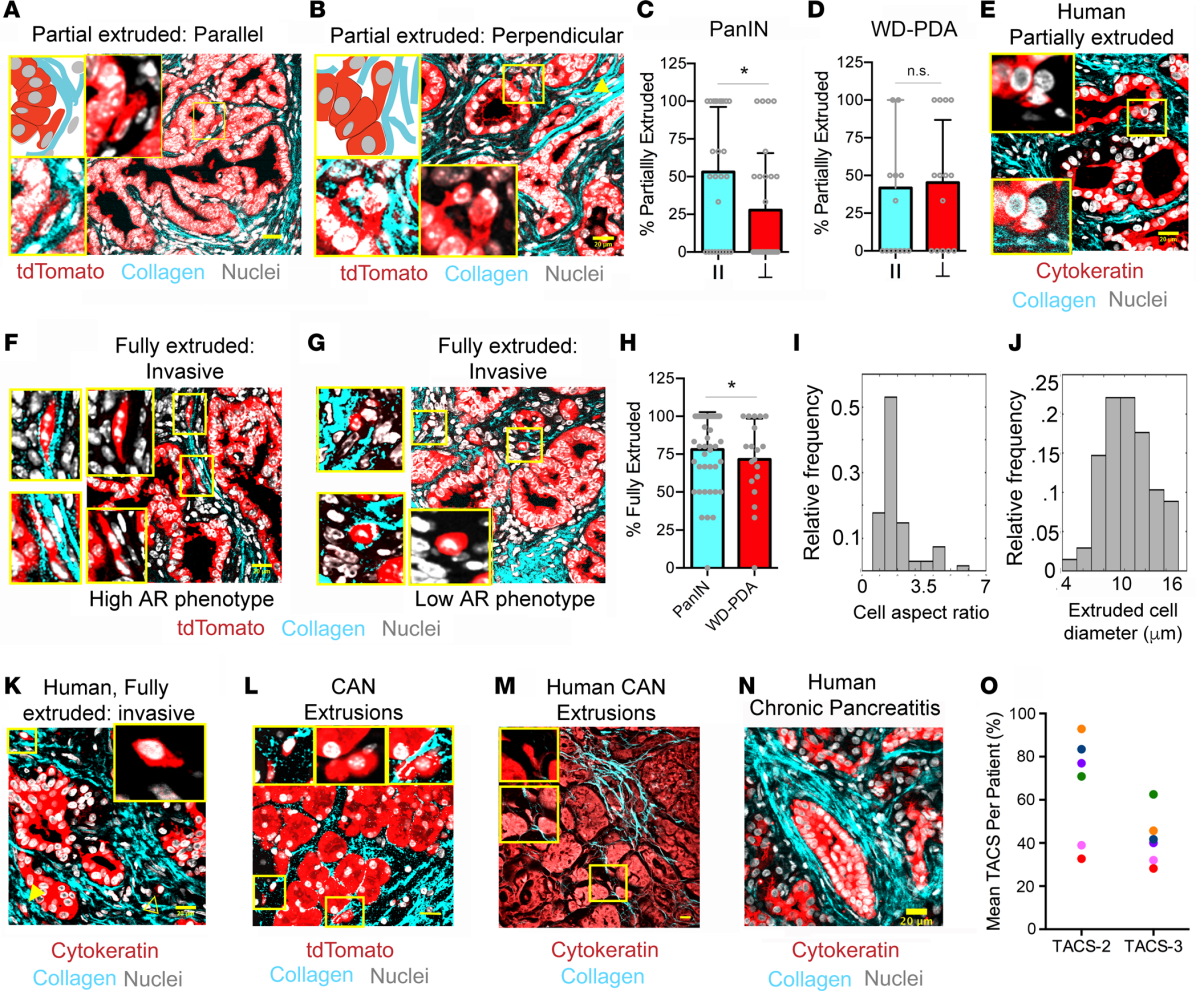
PDA cell extrusion into periductal collagen architectures. (**A** and **B**) Schematic (top left boxes) and micrographs of partial extrusion of carcinoma cells in *KPCT/KPCG* tumor sections into (**A**) TACS-2 and (**B**) TACS-3 in the periductal stroma. Magnified images (yellow boxes) show either all channels (bottom left) or without SHG (insets). Yellow arrowhead (**B**) points to another fully extruded, aligned cell. (**C** and **D**) Frequency of partial cell extrusion in (**C**) PanIN and (**D**) well-differentiated PDA (WD-PDA) associated with parallel TACS-2 (II) or perpendicular TACS-3; data are mean ± SD; *n* = 27 (**C**) and 14 (**D**) FOVs; *P < 0.05 by Mann-Whitney *U* test. (**E**) Partial extrusion in human well-differentiated (WD) PDA samples. Magnified images (yellow box) show either all channels (bottom inset) or without SHG (top inset). (**F**–**H**) Typical fully extruded carcinoma cells (**F** and **G**) in PanIN and well-differentiated PDA and (**H**) their frequency among all extrusions; data are mean ± SD, *n* = 45 (PanIN) and 14 (WD-PDA); *P < 0.05 by Mann-Whitney *U* test. Magnified images (yellow boxes) are shown without SHG (insets) or with all channels (left). (**I** and **J**) Histogram of (**I**) aspect ratios and (**J**) diameters of fully extruded cells in mice; *n* = 68 cells across 12 mouse tumors. (**K**) Fully extruded periductal cells in human WD- PDA samples; Insets show magnified yellow box region, shown without the SHG channel; solid yellow arrowhead (**K**) points to another aligned, fully extruded cell, and outlined yellow arrowhead points to a rounded, nonaligned cell. (**L** and **M**) Cancer-adjacent “normal” regions from *KPC* mice or humans. Insets show magnified images of extruded cells marked with yellow boxes, with all channels or without SHG. (**N**) Collagen architecture surrounding ducts in human chronic pancreatitis (CP). (**O**) Frequency of TACS-2 and TACS-3 in association with ductal structures in human CP (*n* = 8–33 fields of view from 6 patient samples). Scale bars: 20 μm.

Rhim and colleagues (11) demonstrated that invasive dissemination can begin early in PDA development, even prior to detection of histologically malignant disease and frank tumor formation, resulting in single cells in the tumor stroma and DCCs in the blood of 8-week-old *KPC* mice (11). This is strikingly consistent with the timeline of robust collagen deposition and establishment of TACS architectures observed here, and it therefore led us to investigate whether such collagen patterns are key enablers of early dissemination. Furthermore, we note that extrusion of cells from the epithelium and into the lumen (i.e., apical extrusion) is a normal process for epithelium turnover, where apically extruded cells undergo apoptosis via anoikis (32, 33). However, basal extrusion has also been described in normal (32) as well as malignant (33, 34) ductal development and maintenance, and they would precede invasion through the stroma for early dissemination from histologically preinvasive lesions. Therefore, to characterize carcinoma cell extrusion from ductal structures, we performed combined MPE and SHG imaging over multiple regions in several pancreatic tumor sections from a number of *KPCT* and *KPCG* mice. Using ZsGreen1 or tdTomato to identify carcinoma cells (from *KPCG* and *KPCT* mice, respectively) colocalized with a nuclear stain and SHG for collagen, we identified different stages of extrusion associated with distinct collagen patterns. Partially disseminated cells were found extensively around ductal structures in PanIN lesions, and well-differentiated PDA and were, perhaps surprisingly, associated with both TACS-2–like structures and TACS-3 (Figure 2, A and B). Strikingly, these partially disseminated cells at the ductal boundary, while still attached to the main duct, were aligned in the direction of local collagen organization (Supplemental Figure 2, A and C) — i.e., demonstrating contact guidance (10, 28). For PanIN lesions, ~53% of the partially extruded cells, on average, were parallel, and about 28% were perpendicular to the ductal boundary (Figure 2C); they were associated with pancreatic TACS-2 or TACS-3 periductal collagen fiber architectures, respectively. This demonstrates that pancreatic carcinoma cells can enter the less constrained (i.e., looser than the tighter TACS-2 architectures observed in mammary carcinomas; ref. 20) TACS-2 architectures observed in PDA and likely undergo directed motility along the collagen around the ductal structure. In contrast to early disease, partially extruded cells were equally likely to be on TACS-2 or TACS-3 architectures in advanced well-differentiated disease (Figure 2D). This difference is likely the result of the increased frequency of TACS-3^+^ ductal structures in more advanced disease as compared with early PanIN lesions (Figure 1, L and O). Importantly, similar partial dissemination associated with periductal collagen was observed in well-differentiated ductal regions of human PDA sections, where a pan-cytokeratin stain was utilized to visualize carcinoma cells (Figure 2E). Such partially delaminated cells likely represent the early stages of extrusion, and their alignment with the surrounding collagen is indicative of the close association between the ductal epithelium and periductal collagen during this process.

In addition to partial extrusion, we observed an abundance of fully extruded, invasive cells in the periductal space of PanIN lesions and well-differentiated PDA (Figure 2, F–H, and Supplemental Figure 2, A and B), consistent with previous reports identifying carcinoma cells in the stroma during early disease (11, 35) and with carcinoma cells principally following collagen aligned architectures due to contact guidance (Figure 2 and Supplemental Figure 2A). To confirm these findings, we imaged live tumors from *KPCT* mice with combined MPE and SHG microscopy, which also allowed for *Z* stack imaging around dispersed cells to confirm the presence of extruded invasive cells (Supplemental Figure 2C). Consistent with findings in fixed samples, extruded cells were present, and the majority aligned along TACS-2 and TACS-3 architectures, demonstrating contact guidance (Supplemental Figure 2C) and demonstrating that TACS-2 regions often connect to an adjacent TACS-3 region, providing a conduit to migrate away from the duct, while following the ECM alignment (Supplemental Figure 2D). Furthermore, quantitative analysis of the phenotype of fully extruded single cells in the periductal space, which make up the majority of extrusion events recorded (Figure 2H), demonstrated that invading cells assume 2 distinct morphologies. While the majority (~70%) of the cells are elongated and aligned to the orientation of the periductal collagen (Figure 2F), a distinct proportion of more rounded, single cells also exist along collagen in the stroma (Figure 2G). This is further supported by morphological analysis revealing a large range of aspect ratios (AR) and small cell diameters for fully extruded cells (Figure 2, I and J). A significant proportion of cells (~30%) indeed had an AR of less than 1.5 and likely represent a distinct phenotypic population in addition to the elongated, more phenotypically mesenchymal-like single cells, which is consistent with previous observations that heterogeneous cancer cell phenotypes with significant plasticity exist in breast carcinoma and PDA and undergo 3D migration by contact guidance (10, 11, 27, 36, 37). Moreover, these 2 separate phenotypes can be observed in fully extruded cells in patient samples, often existing in the same local region (Figure 2K), further supporting that analysis of cell extrusion in the *KPC* model is highly relevant to human disease and suggesting that phenotypically distinct contact-guided subpopulations are capable of invasion leading to metastasis in PDA.

In contrast to normal tissues that have very little to no collagen around acinar cells and where we did not observe any basal extrusions in either murine or human pancreata (Supplemental Figure 2B), and similar to PanIN lesions and well-differentiated PDA, extrusion events were present in CAN regions adjacent to disease in murine and human tissue, albeit at a lower frequency than observed with PanINs (Figure 2, L and M, and Supplemental Figure 2B). It is striking that, even in CAN regions, which have lower collagen content than PanIN lesions, disseminated cells were almost always associated with regions of periacinar or periductal fibrous collagen (Figure 2, L and M). This suggests that the fibroinflammatory response occurring in tissue directly adjacent to disease may aid the extrusion process, priming the stroma in adjacent regions to facilitate disease spread prior to infiltration of frank malignant disease into these regions. Therefore, we explored TACS architectures in pancreatitis, which represents a strong risk factor for development of PDA and can be a precursor to malignancy (38). Importantly, pancreatitis can display a PDA-like desmoplastic response with an accumulation of straight and thick collagen fibers (23). Indeed, analysis of samples from 6 patients with chronic pancreatitis revealed an abundance of TACS-2 and TACS-3 associated with ductal structures (analysis from 8–33 fields of view from 2 biopsies per patient; Figure 2, N and O). Consistent with our findings in pancreas cancer, approximately 32%–93% of ductal structures were TACS-2^+^ and approximately 28%–62% were positive for both TACS-2 and TACS-3 (similar to pancreatic cancer, ducts that are TACS-3^+^ are also TACS-2^+^ in different regions of the duct; Figure 2O). Remarkably, every pancreatitis patient sample presented with ducts that were TACS-3^+^. However, extrusion events were only infrequently observed relative to findings in PanIN lesions and well-differentiated PDA. This suggests that transformed carcinoma cells are more robustly primed to basally extrude when presented with aligned ECM architectures, as compared to untransformed epithelial cells, or that the desmoplastic phenotype is not as robust as malignant regions, or both. This raises important questions regarding the cooperative cell-intrinsic and -extrinsic mechanisms governing dissemination. Moreover, these data indicate that fibroinflammatory diseases that may precede PDA, such as pancreatitis, can result in stromal ECM architectures primed to facilitate disease spread from the earliest onset of disease.

### Dynamics of single-cell extrusion and contact guided migration in stromal collagen.

**Figure 3:**
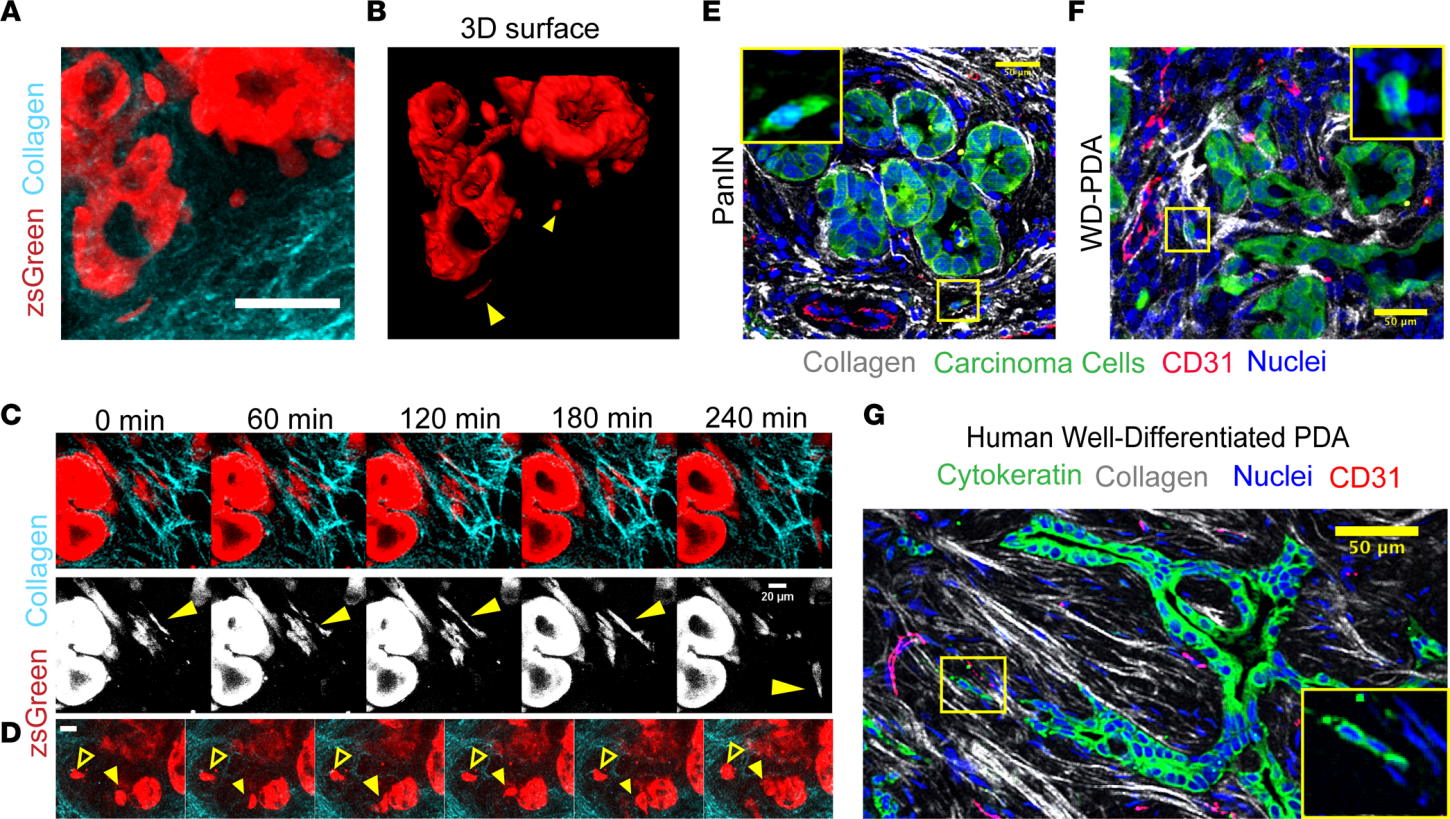
Live imaging reveals dynamics of cell extrusion and invasion in early stage disease. (**A** and **B**) Live 3D imaging of PanIN lesions in a *KPCG* mouse showing extruded cells invading through organized collagen in the periductal space by (**A**) a maximum intensity projection of ~100 μm depth and (**B**) 3D surface rendering of the same region demonstrating that the extruded cells observed in tissue slices were not attached to the main duct at other panels; yellow arrowheads point to extruded cells — see also Supplemental Video 1. (**C**) Time-lapse montage from tracking single, aligned cells in periductal collagen by live MPE/SHG imaging of a *KPCG* tumor; yellow arrowhead indicates cell of interest that migrates rapidly along a collagen fiber (see also Supplemental Video 4). (**D**) Time-lapse montage showing the dynamics of a partially and fully extruded cell in the same field of view in a *KPCG* tumor; yellow solid arrowhead points to a partially extruded cell, still connected to the underlying ductal structure, and yellow outline arrowhead points to a fully extruded cell in the stroma (see also Supplemental Video 5). (**E** and **F**) Immunofluorescence micrograph of *KPCT* tumor sections stained with RFP (shown in green), CD31 (red), and DRAQ5 (blue), demonstrating single extruded cells interacting with aligned periductal collagen (white) directed to blood vessels in (**E**) PanIN lesions and (**F**) well-differentiated PDA; inset shows magnified region (yellow box) displaying the fluorescent reporter and nuclei channels. (**G**) Fluorescence micrograph of human PDA stained with CD31 (red), DRAQ5 (blue), and cytokeratin (green) showing aligned extruded cells following collagen tracks (white) leading to a blood vessel; inset shows magnified region (yellow box) displaying only the fluorescent reporter and nuclei channels. Scale bars: 20 μm (**C** and **D**) and 50 μm (**A**, **B**, and **E**–**G**).

Given the role periductal collagen plays in facilitating basal cellular extrusion, we speculated that TACS located distal to ductal structures also influences cancer cell dissemination through the stroma. Indeed, fully extruded cells in both murine and human samples were found to be colocalized with aligned collagen (Figure 2, F–H and K, and Supplemental Figure 2, A and C), suggesting that TACS not only aid in the dispersal of these cells from the epithelium during basal extrusions, but also guide the extruded cells as they invade through the stroma. Indeed, we have previously demonstrated that carcinoma cells, including those of the pancreas, are strongly directed to migrate along aligned ECM via contact guidance with minimal motility lateral to ECM fiber alignment (10, 22, 27–29). Live MPE/SHG imaging of pancreatic tumor slices again confirmed the prevalence of extruded cells in the periductal space around both PanIN lesions and in well-differentiated PDA (Figure 3A and Supplemental Figure 2). Moreover, to further confirm that the features observed were not an artifact of tissue slicing (i.e., the extruded cells were indeed discrete entities and not part of a larger ductal network connected in other planes), we reconstructed the 3D volume around entire ductal structures using multiphoton imaging of live early disease tumor samples, and we again confirmed the existence of single carcinoma cells in the stroma (Figure 3B and Supplemental Video 1). Interestingly, long-term imaging of ductal structures also revealed dynamic rearrangement and rotational movement of the epithelium, a phenomenon known as coherent angular motion (Supplemental Video 2), which has been previously observed in vitro in mammary acini (39, 40) and is thought to be sustained, in part, by cell division (40). Our observation of this phenomenon in live premalignant and malignant tissue further demonstrates the dynamic nature of epithelial cells in organized ductal structures. These cellular dynamics are perhaps intricately tied to the process of cell extrusion as a mechanism to maintain epithelial tissue homeostasis in the face of aberrant cell division, as in cancers (33).

Furthermore, consistent with static imaging, analysis of live cell dynamics revealed motile cells that have invaded into the stroma. We observed a minor population that are directed by collagen architecture and showed transient switching between more rounded and more elongated phenotypes (Supplemental Video 3), in line with the cell AR analysis (Figure 2). Concurrently, we also observed the larger population of highly elongated cells (i.e., high AR) associated with aligned periductal collagen, rapidly migrating along the collagen tracks (Figure 3C and Supplemental Video 4). We note that these 2 populations are reminiscent of the distinct phenotypes and plasticity that have been separately associated with invasion and metastasis in PDA (11, 36, 37); they are also reminiscent of the contact guidance response of breast carcinoma cells, where we observed a spectrum of phenotypic heterogeneity, from epithelial to those that have undergone epithelial-to-mesenchymal transition (EMT), and robust phenotypic switching (10, 27). Furthermore, through live imaging, we observed cell extrusion from a ductal structure alongside already-extruded cancer cells in the periductal stroma (Figure 3D and Supplemental Videos 4 and 5), providing additional compelling evidence that demonstrates basal extrusions from ductal structures in PDA. Thus, the combined data suggest the potential for a consistent stream of premetastatic dissemination from ductal epithelium, which may not be solely dependent on a stable EMT phenotype but may inevitably contribute to the early metastatic spreading associated with pancreatic cancer.

In line with our conclusions that early extrusion events leading to invasive cells in the stroma contribute to early metastatic spread, we have noted that reports indicate that PDA carcinoma cells can be present in circulation during histologically preinvasive disease (11) and that, in other desmoplastic systems such as in breast carcinoma, pioneer metastatic cells utilize bundles of aligned collagen as highways to escape toward blood vessels (41), often further guided by macrophages (42) in the tumor stroma. Therefore, we visualized the localization of TACS and extruded cells with respect to CD31^+^ blood vessels in archival tissue samples. Carcinoma cells in the stroma were frequently observed in close association with blood vessels in the stroma (Figure 3, E–G, and Supplemental Figure 3). In *KPC* mice, extruded single cells were present on aligned collagen fibers leading to open blood vessels (which are less frequent relative to the larger fraction of collapsed vessels in PDA; refs. 5, 6, 43) in PanIN lesions as well as more mature well-differentiated disease (Figure 3, E and F, and Supplemental Figure 3). Importantly, we confirmed these findings in human tissue samples using pan-cytokeratin to mark cancer cells (Figure 3G). Again, aligned collagen fiber tracks, punctuated with single or streams of multiple carcinoma cells, were observed leading to CD31^+^ blood vessels (Figure 3G and Supplemental Figure 3). It is important to note that, while a large percentage (~75%) of blood vessels in mature PDA are collapsed and nonperfused (5, 6), those in the early stages of the disease have a much higher likelihood of being open (43) and, thereby, likely provide a clearer passage for these constantly extruding cells to escape and enter the bloodstream. Taken together, these data strongly suggest that the phenomenon of early extrusion and invasion along organized collagen fibers plays a direct role in early and extensive metastasis observed in PDA. However, while the intra- and intercellular dynamics during basal cell extrusion from ducts has been explored (33, 34, 44), how the ECM affects this process is, to our knowledge, unexplored, prompting us to delve into its biophysical and molecular mechanisms.

### FAK-dependent mechanotransduction enables collagen-guided cell extrusion and invasion in vitro.

**Figure 4:**
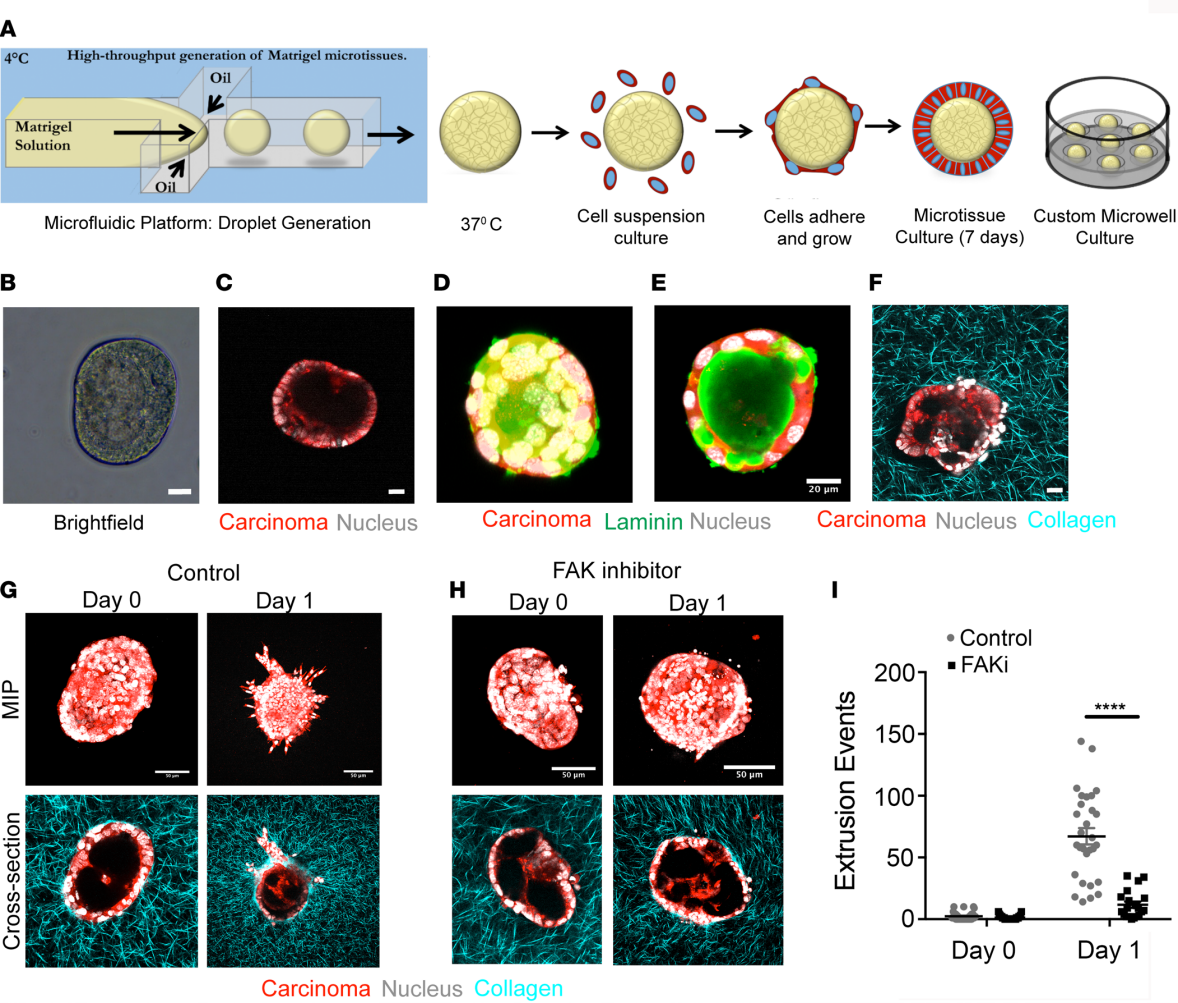
FAK inhibition abrogates collagen fiber–guided PDA cell extrusion in vitro. (**A**) Schematic demonstrating our in vitro platform to mimic tumor cell extrusion from ductal structures into surrounding collagen. Matrigel droplets generated in a microfluidic device by orthogonal flow of Matrigel in an oil based medium, before coating them with primary *KPCG* or *KPCT* cells. Coated droplets are cultured individually in custom-fabricated microwells for several days before embedding in a collagen matrix. (**B** and **C**) Bright-field (**B**) and multiphoton excitation microscopy image (**C**) of a formed microtissue. (**D** and **E**) Maximum intensity projection (MIP) (**D**) and cross-sectional (**E**) MPE immunofluorescence micrographs show a typical droplet structure resembling the cross-section of a duct with basement membrane. (**F**) MPE/SHG imaging of a droplet embedded in collagen matrix. (**G**–**I**) Control (**G**) and FAK inhibitor (**H**) treated conditions, showing significant invasion in the control group at say 1, which is completely abrogated by inhibition of FAK, as quantified by single-cell extrusion analysis in (**I**); *n* = 20–30 droplets per condition from *n* = 2 experiments. Data are mean ± SEM; ****P < 0.0001 by ordinary 2-way ANOVA and Sidak’s multiple-comparison test. Scale bars: 20 μm (**B**–**F**) and 50 μm (**G** and **H**).

The data presented thus far point to periductal collagen architectures as cell-extrinsic drivers of dissemination, but they also indicate that cell-intrinsic properties are likely another key factor guiding single-cell dissemination from ductal structures in PDA, prompting us to explore molecular mechanisms during the cellular response to TACS. Extending our understanding of single-cell contact guidance (10) to multicellular assemblies in ductal structures transitioning to collective or single-cell invasion exposes an interesting duality between cell-cell dynamics and cell-matrix interactions from stromal collagen. A cell at the interface is presented with multiple, counteracting cues to either remain in its cohesive state in the epithelium or break away from its native structure and follow ECM alignment. Indeed, we note that primary PDA cells derived from *KPCG* or *KPCT* mice can display strikingly contrasting morphologies in 3D collagen matrices that have fiber alignments versus 3D Matrigel, the latter representing a more basement membrane–like environment (Supplemental Figure 4A). While cells tended to cluster together in tight epithelial-like structures in Matrigel, they form more tubular, spindle-shaped, protrusive structures in 3D when embedded in a fibrous collagen matrix (Supplemental Figure 4A). This striking difference suggested to us that cells at the interface of a basement membrane–bound epithelial structure and surrounding fibrous collagen matrix experience uniquely counteracting cues that may lead to the extrusion phenotype observed in ductal epithelia in the presence of organized periductal collagen. To dissect this process, we harnessed microfluidic technology to generate primary PDA cell–derived epithelial organoids in a high-throughput system. This allows us to isolate carcinoma cell behavior while retaining key cell and ECM architectures. These organoids could be subsequently embedded into an ECM, whereby epithelial cells encounter similar counteracting cues of cell-cell interactions with basement membrane and aligned collagen (Figure 4, A–F, and Supplemental Figure 4, B and C). Briefly, droplets of Matrigel were generated by an oil-aqueous interface in a microfluidic flow focusing device (Figure 4A) and layered with primary *KPCT or KPCG* cells (Figure 4B). The cells remain organized in a ring around the Matrigel drop, rarely invading into the droplet and resembling a ductal cross-section, as evidenced by 3D imaging (Figure 4, B–E). These microtissues were cultured in individual agarose microwells to allow them to evolve into robust epithelial organoids over several days, where they retain basement membrane (Figure 4, D and E) and are subsequently embedded in matrices where the interaction of individual organoids with the surrounding ECM could be studied (Figure 4F and Supplemental Figure 4, B and C). Indeed, within 24 hours of embedding, both TACS-2 and TACS-3 architectures emerged surrounding the organoids (Supplemental Figure 4D) and cancer cells began to extrude and disseminate along collagen fibers (Figure 4G), where collective invasions and single-cell extrusions to invasion were observed (Supplemental Videos 6–8). In contrast, organoids embedded in Matrigel largely retain a rounded ductal morphology (Supplemental Figure 4, B and C). Importantly, in collagen matrices, the features of extruding regions are highly reminiscent of ductal outgrowths and of collective and single-cell dissemination associated with TACS-3 observed in murine and human PDA samples (Figures 1 and 2, Supplemental Figure 2C, and Supplemental Figure 4E). Furthermore, rapid extrusion led to extremely robust invasion under control conditions over 3 days (Supplemental Figure 4F), consistent with findings with mammary epithelial acini (45). These data indicate that our potentially novel in vitro system, particularly at early time points, can recapitulate key features of the extrusion to invasion process and provides a high-throughput controllable platform in which to investigate the molecular mechanisms guiding epithelial extrusion in PDA.

The fact that embedding these epithelial structures within collagen matrices is sufficient to induce extrusion and invasion is in line with the conclusion that this dynamic process is guided by structural cues in the periductal ECM. In response to this extrinsic factor, we investigated cell-intrinsic signaling that may modulate cell extrusion. The intercellular force generation machinery and the molecular linkages between focal adhesions, myosin, and F-actin — which are key mediators of contact guidance (10, 46, 47) — are likely to be important for extrusion into collagen matrices. Indeed, consistent with this hypothesis, increased matrix density and stiffness promote ECM alignment and mammary cell invasion into 3D matrices through a FAK/ERK signaling axis, where inhibition of FAK function by expression of dominant-negative FRNK reverts the invasive phenotype (17). Moreover, expression of both total and phosphorylated FAK in PDA carcinoma cells is well established in the literature (7, 48–50), and a recent study by Jiang and colleagues demonstrates that targeting FAK in PDA not only augments immunotherapy efficacy, but also decreases the number of single PDA cells observed in the periductal space (7). Thus, this collective evidence prompted us to hypothesize that FAK signaling promotes cell extrusion and subsequent invasion associated with aligned ECM. To test the impact of FAK inhibition (FAKi), we first employed our in vitro epithelial-ECM interface platform. Consistent with our hypothesis, FAKi dramatically reduced cell extrusion, to the extent that very little single-cell extrusion was observed at day 1 in the FAKi group versus robust extrusion in the vehicle control group (Figure 4, G–I; see also comparison between Supplemental Videos 8 and 9). Indeed, we observed robust expression of phosphorylated FAK (pFAK) in invasive carcinoma cells and decreased pFAK following FAKi (Supplemental Figure 4G), although whether or not pFAK may be differentially localized at the regions of early extrusion remains to be defined. This behavior was maintained through day 3, where extrusion and invasion were profoundly limited by FAKi compared with controls (Supplemental Figure 4F). However, we note that in cells lacking sphingosine-1-phosphate (S1P) receptors, FAK has also been suggested to regulate apoptosis associated with basal extrusions in monolayer culture (34). As such, we evaluated apoptosis levels in basally extruded cells to determine if our observed decreases in extrusion and invasion under FAKi could be due, either entirely or in part, to apoptosis. In the primary cells within 3D environments in this study, analysis of cleaved caspase-3 (CC3) revealed no differences in apoptosis between control and FAKi-treated carcinoma cells (Supplemental Figure 4H). Interestingly, concomitant with the decrease in extrusion, we did not observe a reduction in organized collagen, especially TACS-3–like arrangements that were present around the epithelial structures in both control and FAKi groups (Figure 4, G and H, and Supplemental Figure 4D), suggesting that even in the presence of TACS-3-like architectures, inhibition of FAK profoundly reduces the ability of cells to extrude and then invade along aligned ECM. We do, however, observe limited collective invasions in the FAKi group at later time points (days 2–4) where single-cell invasion remains severely impaired (Supplemental Figure 4F), which is consistent with cell proliferation and general motility-related quasiballistic invasion into aligned ECM resulting from cell crowding (51). Thus, our data suggest that FAKi cells still retain limited ability to outgrow from well-defined ductal structures, but disrupting the key signaling node of focal adhesion force transmission and contact guidance by targeting FAK makes single-cell invasion quite inefficient.

### FAKi abrogates single-cell extrusion and metastasis in PDA.

**Figure 5:**
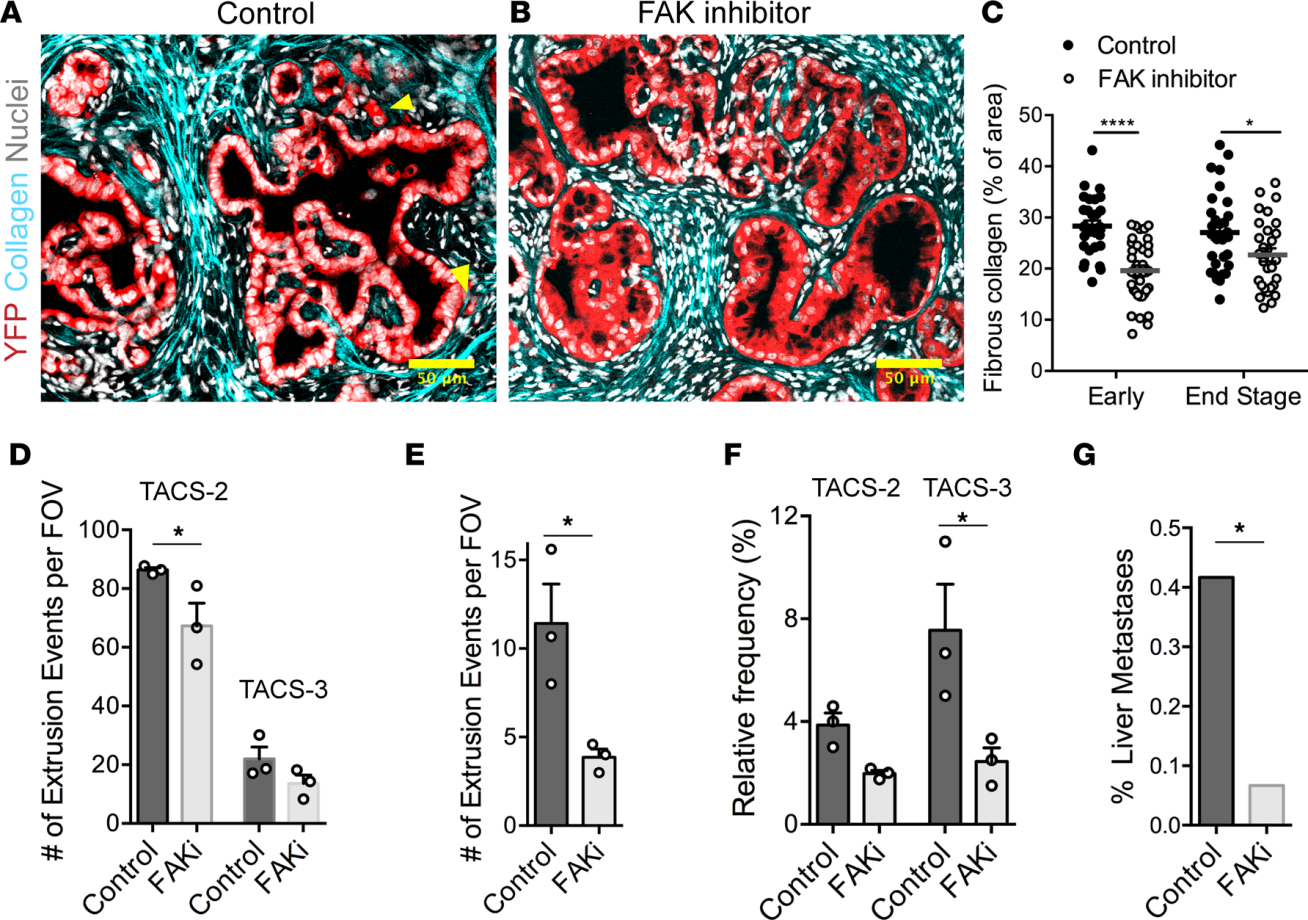
Inhibition of FAK alters frequency of collagen architectures, extrusion, and metastasis in PDA. (**A** and **B**) Fluorescence micrographs of stained *KPCY* sections either treated with (**A**) vehicle control or (**B**) FAK inhibitor showing reduction in collagen deposition around ductal structures, smoother ductal boundaries, and reduction in cell extrusion; yellow arrowheads indicate single-cell extrusion events in the control sample. (**C** and **D**) Quantification of collagen content in *KPC* mice shows a reduction in deposition of fibrous collagen in both early (1.5 month) and end-stage mice, with a (**D**) concomitant reduction in TACS-like architectures in *KPC* mice. Note, the vast majority of PanIN lesions in FAKi-treated mice still retain TACS-2 and TACS-3 architectures in spite of lower overall collagen levels. (**E**) Number of extrusion events quantified from control and FAKi-treated mice showing reduced extrusion by FAK inhibition. (**F**) Extrusion events for the control and FAK inhibited groups quantified as a function of TACS-2 or TACS-3 architectures showing a decrease in extrusion and especially into TACS-3 regions. (**G**) Frequency of liver metastasis in control and FAKi-treated *KPC* mice showing reduction in liver metastases with FAK inhibitor treatment (data are from ref. 7). Data are mean — SEM (**C**–**F**), *n* = 29–36 FOV per group for **C**, *n* = 3 per group for **D**–**F**, and *n* = 6 for **G**. ****P < 0.0001, *P < 0.05 by 2-way ANOVA and Sidak’s multiple-comparison test for **C**, **D**, and **F**; *P < 0.05 for **E** by *t* test; *P < 0.05 by Fisher’s exact test for **G**. Scale bars: 50 μm.

Previous work demonstrated that FAK and pFAK are expressed throughout PanIN lesions, frank PDA, and invasive carcinoma cells in the stroma (7, 48–50), consistent with our finding of continuous pFAK expression throughout PanIN lesions (Supplemental Figure 4I). However, while we note that any potential differential pFAK localization in protrusions from individual extruding cells in vivo is yet to be defined, FAKi leads to a decrease in the number of invasive PDA cells observed in the periductal space (7). We, therefore, analyzed PDA formalin-fixed paraffin-embedded (FFPE) sections from vehicle versus FAKi groups to interrogate cell extrusion and invasion in the context of collagen fiber organization to test our hypothesis that FAK influences early dissemination in vivo. Consistent with the general abrogation of fibrosis, FAKi led to a decrease in fibrillar collagen in both early (1.5-month group) and end-stage *KPC* mice (Figure 5, A–C) with a very modest, yet significant, decrease in TACS-2 architectures and a modest, not significant, trend of less TACS-3 architectures (Figure 5D). Thus, while collagen levels are reduced, likely by attenuation of FAK-dependent mechanotransduction in myofibroblasts that regulates the fibroinflammatory response in PDA (7, 50, 52), TACS architectures are still present in the vast majority of ductal structures (Figure 5, C and D). As such, we can evaluate cell-intrinsic FAK signaling in response to collagen architectures already in place. In agreement with our hypothesis and in vitro data, there was a significant decrease in the frequency of single-cell extrusions following inhibition of FAK (Figure 5E). We also observed smoother and more organized ductal morphology in FAKi-treated mice, indicative of a less advanced or more limited progression of disease (Figure 5, A and B). Interestingly, the number of extrusions associated with both TACS-2 and TACS-3 showed decreasing trends following FAKi. However, the difference was significant and much more pronounced for TACS-3–associated extrusions (Figure 5F), consistent with our in vitro findings, suggesting that TACS-3–mediated extrusions and invasion may be more FAK dependent. This is critical, since collective data suggest that robust dissemination of extruded cells invading through periductal TACS-2 architectures ultimately relies on TACS-3 architectures to serve as an exit point from the duct-adjacent space and that decreased extrusion and invasion would limit metastatic burden. Indeed, *KPC* mice undergoing FAKi treatment from the same cohort showed decreased metastasis to the liver (7) (Figure 5G), consistent with the conclusion that FAK-dependent single-cell extrusion and subsequent invasion through the stroma promotes metastasis. Overall, these data implicate that FAK may be a key mechanotransduction node during the response to established TACS-3–aligned collagen that then influences the frequency of early single-cell extrusion and subsequent invasion in PDA leading to enhanced metastatic dissemination. Thus, early dissemination and, therefore, early metastasis in PDA is driven, at least in part, by both the local organization of periductal collagen and focal adhesion–dependent recognition of these ECM patterns by carcinoma cells.

## Discussion

Our findings represent a key advancement in the understanding of how the desmoplastic ECM drives disease progression in PDA. We utilize combined MPE and SHG imaging to visualize and quantify collagen architectures in PDA and establish critical links between the periductal collagen organization and early invasion in PDA. Indeed, it is clear that the deposition and organization of periductal collagen play important roles in the early dissemination and metastasis of PDA and may be used as biomarkers for disease progression, where TACS^+^ findings around histologically “early” disease may indicate more advanced disease than classic histology would suggest and aid the pathology and treatment plan processes. Furthermore, one can envision using TACS to map and identify key features for tracking disease progression in other carcinomas (53, 54). Overall, these insights offer a bridge between the cell biology of basal epithelial extrusion, invasion through 3D environments, and clinical oncology, and they provide further impetus to the growing paradigm of stroma-targeted therapies to normalize cellular microenvironments in pancreatitis and cancer.

In this work, we identify and map previously established disease-relevant collagen signatures (i.e., TACS in breast tumors) in pancreatic disease. Both TACS-2 and TACS-3 manifest with largely straightened collagen fibers in PDA, albeit often with a looser organization for TACS-2 than was observed in mammary carcinomas. As we observe TACS-2 architecture connecting to TACS-3 regions (Supplemental Figure 2D), we speculate that carcinoma cells extruded into TACS-2 regions migrate along collagen around ducts until encountering a TACS-3 exit point or do not easily leave the periductual space since migration perpendicular to dense collagen is profoundly less efficient and, in many cases, extremely rare (10, 22, 27, 29, 51). Furthermore, we note that collagen is highly anisotropic with a modulus that is orders of magnitude larger in the direction of alignment versus other directions (e.g., lateral deformations, bending, compression) and that widespread straightening of the collagen fibers has been associated with increased stiffness due to the well-established strain-stiffening behavior of fibrillar collagen (55). As such, elevated stiffness in itself has important implications in tumor progression (17, 19, 56), suggesting that the TACS architectures not only contribute to contact guidance, but also likely promote disease progression through stiffness-regulated mechanotransduction signaling. Likewise, these straightened collagen networks are also consistent with increased tumor pressures and poor drug transport observed in PDA, as well as the previous descriptions that hyaluronan-driven swelling pressures are constrained by a stressed collagen network in PDA (5, 57). Furthermore, we observed cell extrusions in cancer-adjacent normal sections showing signs of inflammation and collagen deposition. One could posit that this acts to prime the adjacent regions for disease infiltration and simultaneous entrance into the metastatic cascade. Indeed, this development of the ECM phenotype may precede and even drive the malignant phenotype in these adjacent regions. Importantly, our findings suggest that this is likely the case for fibrotic diseases like pancreatitis. Notably, the onset of induced pancreatitis appears to augment early dissemination in *KC* and *KPC* mice (11), and we found ample evidence of fibrotic collagen in the form of TACS-2 and TACS-3 in human pancreatitis, but with minimal extrusion (Figure 2), suggesting that TACS alone may not be sufficient for robust cell extrusion in untransformed cells. Therefore, the risk factor posed by chronic pancreatitis for malignant transformation may at least, in part, be attributed to the generation of the ECM phenotype as a priming microenvironment for PDA to arise and disseminate from early stages. This suggests that therapeutic strategies to resolve fibroinflammation, such as stroma-targeting antifibrotic therapies (4, 43), may be beneficial in disrupting ECM architectures conducive to disease spread, thus impeding development of early DCCs in patients who later develop PDA.

In terms of the cell-intrinsic pathways regulating the cell biology of basal extrusion from ductal epithelia, data suggest that S1P and Rho-mediated signaling play a role mediating actomyosin contractility during extrusion of epithelial cells in monolayer culture conditions (34). Likewise, loss of p120 catenin in *Kras*-driven PDA also promotes epithelial cell extrusion with concomitant increases in fibrotic stroma in early disease in *KC^iMst1^* mice (35), and this is consistent with our findings of stromal architecture guiding basal extrusions and invasion. Here, we also show that, in PDA, FAK is crucial for robust cell extrusion and subsequent invasion through organized ECM. Notably while, in vivo, the FAK inhibitor likely affects other stromal cells and modulates their behavior (e.g., reducing ECM production by myofibroblasts), our in vitro system allows us to test the effect of the inhibitor specifically on primary carcinoma cells. In addition, although FAK is known to play many regulatory roles (which the inhibitor can alter), our combined data suggest that its role as a mechanotransduction node at the cell-ECM interface during cell motility may be most pertinent here. Indeed, recent work has shown that, in stiff environments, contact guidance is orchestrated by anisotropic forces arising from constrained focal adhesion maturation on discrete collagen fibers and that intercellular forces can counteract such directional cell-ECM forces (10, 47). A corollary to this theory suggests that a carcinoma cell interacting with organized periductal collagen may experience sufficient anisotropic forces to overcome strong intercellular forces and extrude out of the epithelium. Thus, basal extrusion is clearly a biophysical process requiring force imbalance, not dissimilar to the anisotropic forces driving contact guidance and cell-cell forces that can compete with cell-ECM forces (10). Taken together, our data suggest that this principle may be applied generally to study cell-ECM interactions and that the balance of cell-cell and cell-ECM forces is a key factor driving cell extrusion, as well as single- and collective-cell invasion and colonization. Along these lines, here, we introduced a microfluidic-based high-throughput assay that enables the study of delamination of carcinoma cells at the epithelial-collagen interface. This system provides a highly controllable platform to model the epithelial-ECM interface and can be used in the future to address fundamental developmental and disease biology and perform rapid drug screening. Certainly, it could be used to support additional studies to further establish and define focal adhesion-cytoskeleton-contractility–related pathways necessary for fundamental extrusion and motility process and to determine the differential effects of various stromal components in this process, which are much more readily controllable in vitro than in vivo.

In terms of therapy, our findings provide further evidence to support the development of stroma-targeting therapies (STTs) to treat desmoplastic diseases like PDA (4, 5, 7, 43). Indeed, we and others have recently developed regimens to target the stroma in autochthonous-established PDA (5, 7, 8, 43) to provide strong consensus evidence of the potential for disrupting the stroma. For instance, we recently demonstrated that halofuginone can help normalize carcinoma-associated fibroblast (CAF) behavior to robustly decrease the fibrotic response in PDA while also increasing antitumor immunity (43). Likewise, antifibrotic therapy with losartan has been shown to decrease fibrous ECM and has gone through promising Phase II trials (58), and multiple early trials with FAK inhibitors are ongoing for PDA (e.g., NCT02758587, NCT04331041, NCT03727880; www.clinicaltrials.gov). Thus, therapeutic strategies to reengineer TMEs to move them toward normalization may have a role not only in limiting continued dissemination from unresectable invasive disease during treatment, but also in very early disease. As much needed approaches for early PDA detection advance, it may be necessary to couple stromal and molecular therapies (such as STT and FAKi approaches) for early treatment in order to limit early dissemination. We note that this dissemination program is prevalent through all stages of disease, actively and continually promoting disease spread, suggesting that disrupting this process may be a viable strategy to slow or halt disease progression during therapeutic interventions to combat already-established primary and metastatic disease. Likewise, it may be beneficial to employ STT strategies to treat precursor diseases such as pancreatitis. We observed robust fibroinflammatory activity in pancreatitis with establishment of ECM that results in stromal architectures that are primed for robust dissemination prior to transformation such that they can facilitate disease spread from the earliest onset of disease. Removing these architectures in at-risk patients may be a strategy to impede early dissemination in persons who later develop PDA. Indeed, stroma-normalizing approaches could be explored as a prophylactic drug to dismantle the microenvironment that is likely to promote disease progression and early metastasis.

## Methods

Supplemental Methods are available online with this article.

### Human and mouse pancreatic tissues and tumors.

Normal and diseased pancreatic samples (PDA and chronic pancreatitis) from patients were obtained as FFPE sections in the form of a tissue with associated pathology information (staging, grading, etc.; US Biomax Inc). In addition, freshly resected PDA, CAN, and chronic pancreatitis sections from the clinic were obtained from BioNet (University of Minnesota) in compliance with approved IRB protocols. Fresh tissues were kept on ice in cell culture media for transportation, formalin-fixed immediately upon arrival, and subsequently paraffin-embedded and sectioned for analysis.

Genetically engineered *KPC* mice were used as a faithful mouse model for pancreas cancer that recapitulate key stromal dynamics and metastatic spread observed in human disease (5, 24). Pancreatic tumor samples were obtained from variants of the *KPC* model (*KPCT*, *KPCG,* or *KPCY* [*ROSA26^LSL-YFP^*]) with fluorophores expressed specifically in pancreatic carcinoma cells.

### Cell culture.

Primary PDA cell lines were derived from pancreatic tumors in *KPCG* or *KPCT* mice, and they were derived as previously described (5). Following establishment and purification, cells where maintained in high-glucose DMEM supplemented with 10% FBS. Three-dimensional culture was conducted as we described previously (17, 27). Briefly, these primary pancreatic carcinoma cells were embedded in 3 mg/mL collagen-I (Corning) matrices or in Matrigel (Corning), diluted in complete medium to a concentration of 4 mg/mL, for 2 days before imaging (see “Engineering microtissues to analyze cell extrusion and invasion” in Supplemental Methods).

### Quantification of collagen content and architecture.

For unbiased quantification of collagen content and TACS, multiple SHG images were taken at random locations on several slides obtained from different *KPCT* or *KPCG* mice or patients. These images of different regions represented various grades or stages of PDA progression, such as normal, adjacent normal, PanIN, undifferentiated PDA, or well-differentiated PDA, which were determined by combining pathological assessment (also in case of the human samples) and inspection of H&E stained serial sections of the same tumor. The SHG images were then run through a custom Matlab code that dynamically thresholds images to retain collagen positive pixels in a binary image. The percentage of positive pixels was then calculated and used as a measure of fibrous collagen content. Quantification of TACS-2 and TACS-3 was obtained using CurveAlign (LOCI), a freely available software that calculates the angular distribution of collagen fibers with respect to a user-defined ductal boundary (59). To obtain the frequencies of TACS-2 and TACS-3 occurrence, entire millimeter-scale regions of the human samples were imaged using MPE and SHG, and they were reconstructed; the number of total ducts and the number of TACS-2^+^ and TACS-3^+^ ducts were noted, and corresponding percentages were obtained.

### Live cell imaging of tumor explants.

Freshly excised tumor slices were obtained from *KPCT* and *KPCG* mice. Live cell imaging of tumor slices was performed similar to time-lapse MPE methods described previously (27). Briefly, the tumor was sliced into a few thin sections (300–350 µm) using a vibratome, set on a 35 mm dish with a slice anchor (Warner Instruments), and overlaid with L-15 media supplemented with 10% FBS, penicillin-streptomycin, plasmocin, fungizone, and 10 μg/mL soybean trypsin inhibitor (MilliporeSigma). Imaging was subsequently performed on the multiphoton microscopy setup described above with a custom-built temperature-controlled stage insert (60) for 6–12 hours with a time interval of 20 minutes between frames at 880–900 nm excitation wavelength and emission captured in the green (*KPCG*), red (*KPCT*), or blue (SHG) channels. Analysis and 3D rendering of *Z* stacks, visualization of time-lapse imaging data, and image processing were done in Fiji. For live imaging, *Z* stacks were generally obtained at a step size of 4–5 μm, covering a total depth of about 80–100 μm.

### Analysis of cell extrusion in vivo.

For analysis of cell extrusion, unbiased images of CAN, PanIN, and differentiated PDA regions (17–54 fields of view/group) were obtained from pancreatic tumor sections (6–13/group) from different *KPCT* or *KPCG* mice (7–9 mice/group). Within each field of view (300 μm × 300 μm or 600 μm × 600 μm), the number of ductal structures, the percentage with organized periductal collagen (either TACS-2 or TACS-3), and the total number of extrusion events were calculated. An extrusion event was defined as a single (or dividing) disseminated cell in the periductal stroma (fully extruded) or a single cells or pair of cells protruding from the smooth boundary of a duct (partially extruded). The association of partially extruded cells with TACS-2^+^ or TACS-3^+^ areas or those of fully extruded cells with aligned collagen in the stroma was determined by visual inspection, keeping in mind quantitative measures described in Figure 1. Morphometric analysis of disseminated cells was performed manually in Fiji, including only single cells fully extruded into the stroma for the analysis.

### FAKi in KPC mice.

Control and FAKi samples have been previously described (7). Briefly, 50 mg/kg VS-4718 was administered by oral gavage twice a day in a formulation with 0.5% carboxymethyl cellulose and 0.1% Tween-80 (MilliporeSigma) in sterile inhibitor.

### Statistics.

Multiple groups were compared by ANOVA (2-way for 2 or more groups of 2 factors, and 1-way for a single variable of more than 2 factors), followed by the Tukey’s post hoc analysis, or the nonparametric Kruskal-Wallis test with Dunn’s post hoc testing, as dictated by the size and distribution of the data. Similarly, 2-way ANOVA with Sidak’s multiple-comparison test was used to compare multiple groups across different conditions. The nonparametric Mann Whitney *U* test or 2-tailed Student’s *t* test was employed for testing null hypotheses between 2 groups. Number of data points for each experiment, the specific statistical tests, and significance levels (*P* < 0.05 was considered significant) are noted in the figure text.

### Study approval.

All animal studies were approved by the IACUC of the University of Minnesota. All human samples were deidentified and obtained either through the UMN BioNet Shared Resource program at the University of Minnesota in accordance with University of Minnesota IRB approval that includes informed consent for tissue donation or from publically available commercial sources.

## Author contributions

PPP initially conceptualized the study. AR, MKC, and PPP participated in the design of the study. AR, MKC, NJRM, HRR, ALC, HJ, DGD, DKW, and PPP participated in the design of experiments. AR, MKC, NJRM, ALC, MC, KBE, EAE, JHS, HRR, AAK, HJ, and PPP generated unique reagents and performed experiments and/or analyses. AR developed quantitative metrics and algorithms. ALC and DKW developed the microfluidic platform and developed its use here with AR, MKC, and PPP. HJ and DGD generated murine tissues from FAKi studies and assisted in experimental design and data interpretation. PPP and DGD secured funding. AR, MKC, and PPP wrote the manuscript, with significant edits by NJRM. Authorship order of co–first authors was determined on the basis of who started work on the project first. All authors read and contributed comments to the final manuscript. PPP oversaw all aspects of the study.

## Supplementary Material

Supplemental data

Supplemental video 1

Supplemental video 2

Supplemental video 3

Supplemental video 4

Supplemental video 5

Supplemental video 6

Supplemental video 7

Supplemental video 8

Supplemental video 9

## Version 1 12/16/2021

In-Press Preview

## Version 2 02/08/2022

Electronic publication
