# Supplementary material for: Stromal architecture directs early dissemination in pancreatic ductal adenocarcinoma
Source: JCI Insight. 2022 Feb 8;7(3):e150330. doi: 10.1172/jci.insight.150330 (PMC8855836; doi:10.1172/jci.insight.150330)
Supplement: Supplemental data [file jciinsight-7-150330-s137.pdf]

Supplementary Information

Stromal architecture directs early dissemination in pancreatic ductal adenocarcinoma

Arja Ray[1,2*], Mackenzie K. Callaway[1,2*], Nelson J. Rodríguez-Merced[1,2], Alexandra L. Crampton[1,2], Marjorie Carlson[1,2], Kenneth B. Emme[1,2], Ethan A. Ensminger[1,2], Alexander A. Kinne[1], Jonathan H. Schrope[1], Haley R. Rasmussen[1], Hong Jiang[3], David G. Denardo[3], David K. Wood[1,2], Paolo P. Provenzano[1,2-4-6#]

Affiliations:

[1] Department of Biomedical Engineering, University of Minnesota
[2] University of Minnesota Physical Sciences in Oncology Center
[3] Department of Pathology and Immunology, Washington University School of Medicine
[4]Masonic Cancer Center, University of Minnesota
[5] Institute for Engineering in Medicine, University of Minnesota
[6] Stem Cell Institute, University of Minnesota

*These authors contributed equally
#To whom correspondence should be addressed: pprovenz@umn.edu

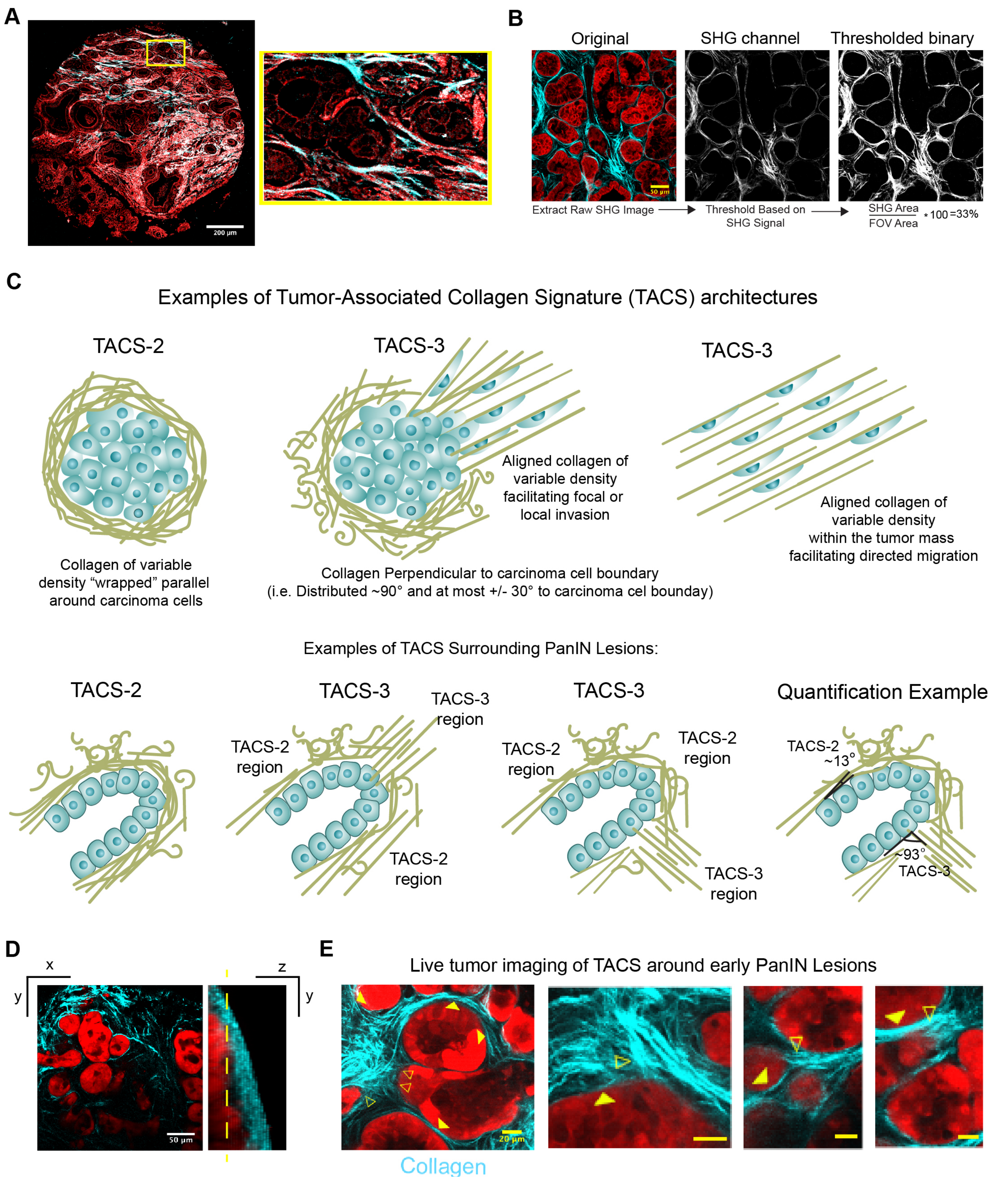

Supplementary Fig. 1: Imaging and analysis of collagen patterns in human and murine PDA: (**A**) Imaging and quantification of whole human biopsy samples (from TMAs with associated pathology staging and grading) for quantification of collagen signatures. Yellow box shows magnified view of the boxed region

showing fibrous collagen patterns around ductal structures. (**B**) Example of fibrous collagen quantification from a *KPCT* sample. (**C**) Examples of Tumor-Associated Collagen Signatures (TACS) and examples of these architectures near PanIN lesions and schematic of quantifying TACS around ductal structures. (**D**) 3D imaging of a PanIN section of a *KPCT* mouse with xy and yz views showing collagen wrapped around ductal structures- yellow dashed line shows the z plane of the xy image (**E**) Live multiphoton excitation of tdTomato expressed in carcinoma cells (*red*) with combined second harmonic generation of collagen (cyan) showing four additional examples of regions of the duct are positive for TACS-2 (solid arrowheads) while adjacent regions of the ducts are positive for TACS-3 (open arrowheads), demonstrating that collagen surrounding “pre-invasive” PanIN lesions possess ECM patterns that are known to promote invasion of pancreatic carcinoma cells. Scale bars = 200 μm (A), 50 μm (B, D), and 20 μm (E).

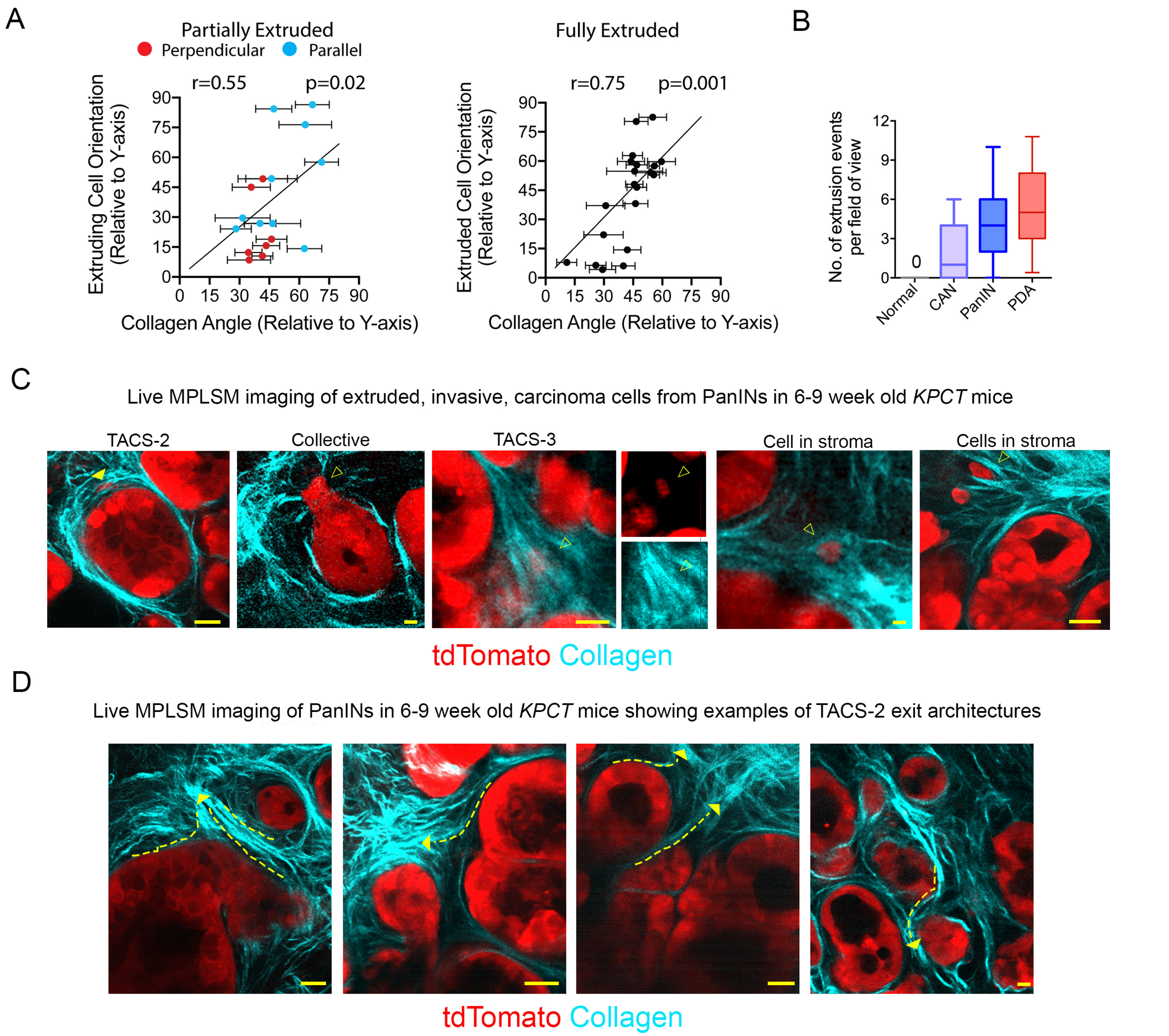

Supplementary Fig. 2: Extruded and invasive cells associated with TACS architectures: (**A**) Correlation between partial or extruded cells and collagen orientation showing associations between cell aspect ratio direction and fiber alignment (n=17, 20, r: Pearson's correlation coefficient, p: p-value for the null hypothesis of no correlation) Data are mean +/- SEM. (**B**) Quantification of extrusion events for normal, CAN, PanIN and well-differentiated PDA from *KPCT* and *KPCG* mice (n=3, 17, 53, 21 samples in each group), box-whisker plots show min to max with median and interquartile range. (**C**) Live tissue multiphoton laser-scanning microscopy (MPLSM, i.e multiphoton excitation of tdTomato expressed in carcinoma cells (*red*) with combined second harmonic generation of collagen (*cyan*)) in young mice demonstrating extrusion events associated with aligned collagen regions. Again, we note that regions of the duct are positive for TACS-2 while adjacent regions of the ducts are positive for TACS-3. (**D**) Live MPE and SHG imaging identifying TACS3 "exit points" from TACS2 regions. Arrows indicate examples of TACS2 to TACS3 paths. Scale bars = 20 μm.

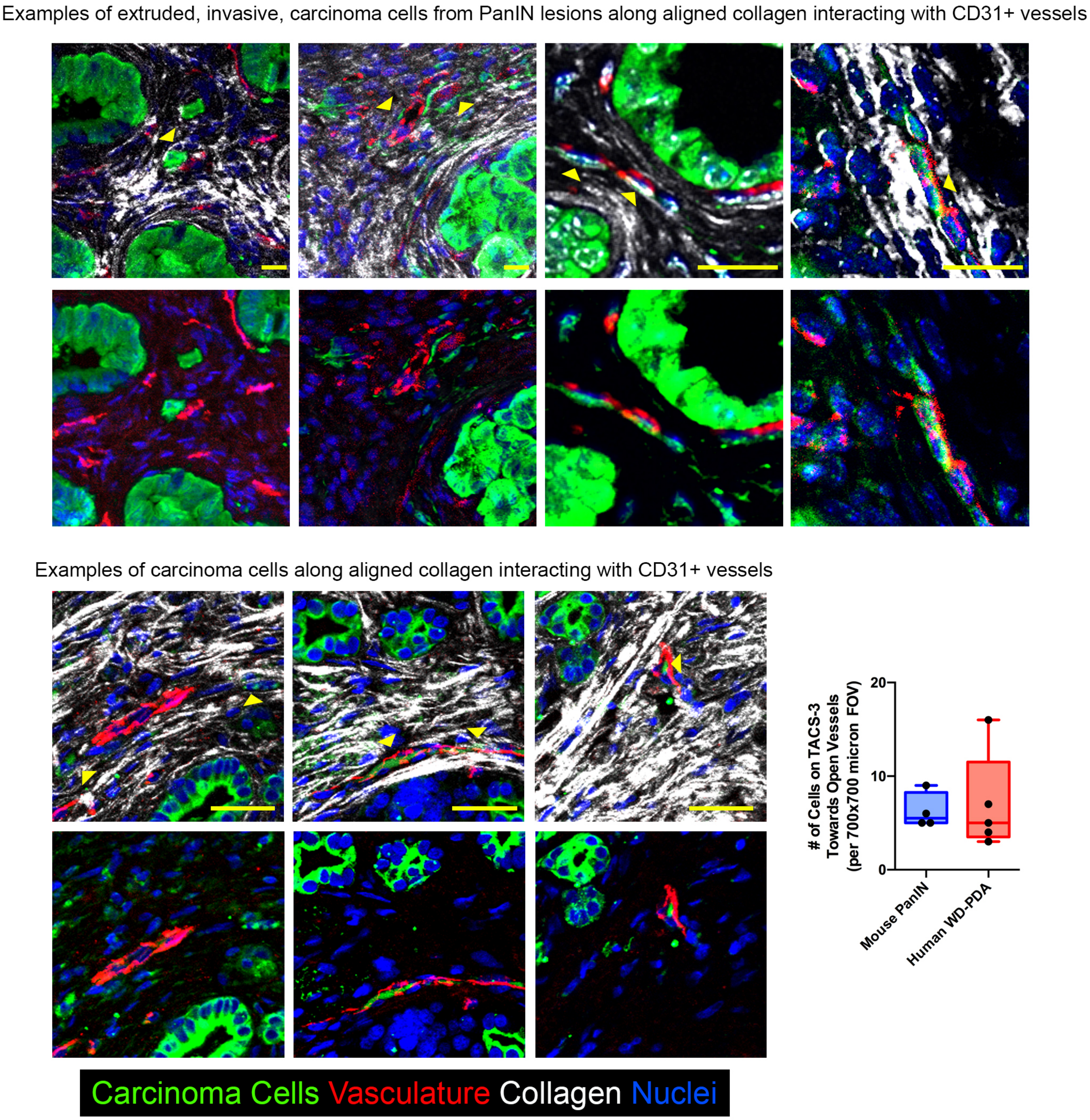

Supplementary Fig. 3: Extruded cell invading along aligned collagen to blood vessels. (***Top***) Immunofluorescence micrographs of *KPCT* tumor sections stained with RFP (*shown in green*), CD31 (*red*), and DRAQ5 (*blue*) demonstrating single extruded cells interacting with aligned periductal collagen (*white*) directed to blood vessels associated with early stage PanIN lesions; *Second row* shows immunofluorescence staining without collagen SHG signal. (***Bottom***) Immunofluorescence micrographs of well-differentiated human PDA stained with Cytokeratin (green), CD31 (*red*), and DRAQ5 (blue) demonstrating aligned extruded cells following collagen tracks (*white*) leading to blood vessels; *Fourth row* shows immunofluorescence staining without collagen SHG signal.. Scale bars = 20 μm (*top panels*) and 50 μm (*bottom panels*).

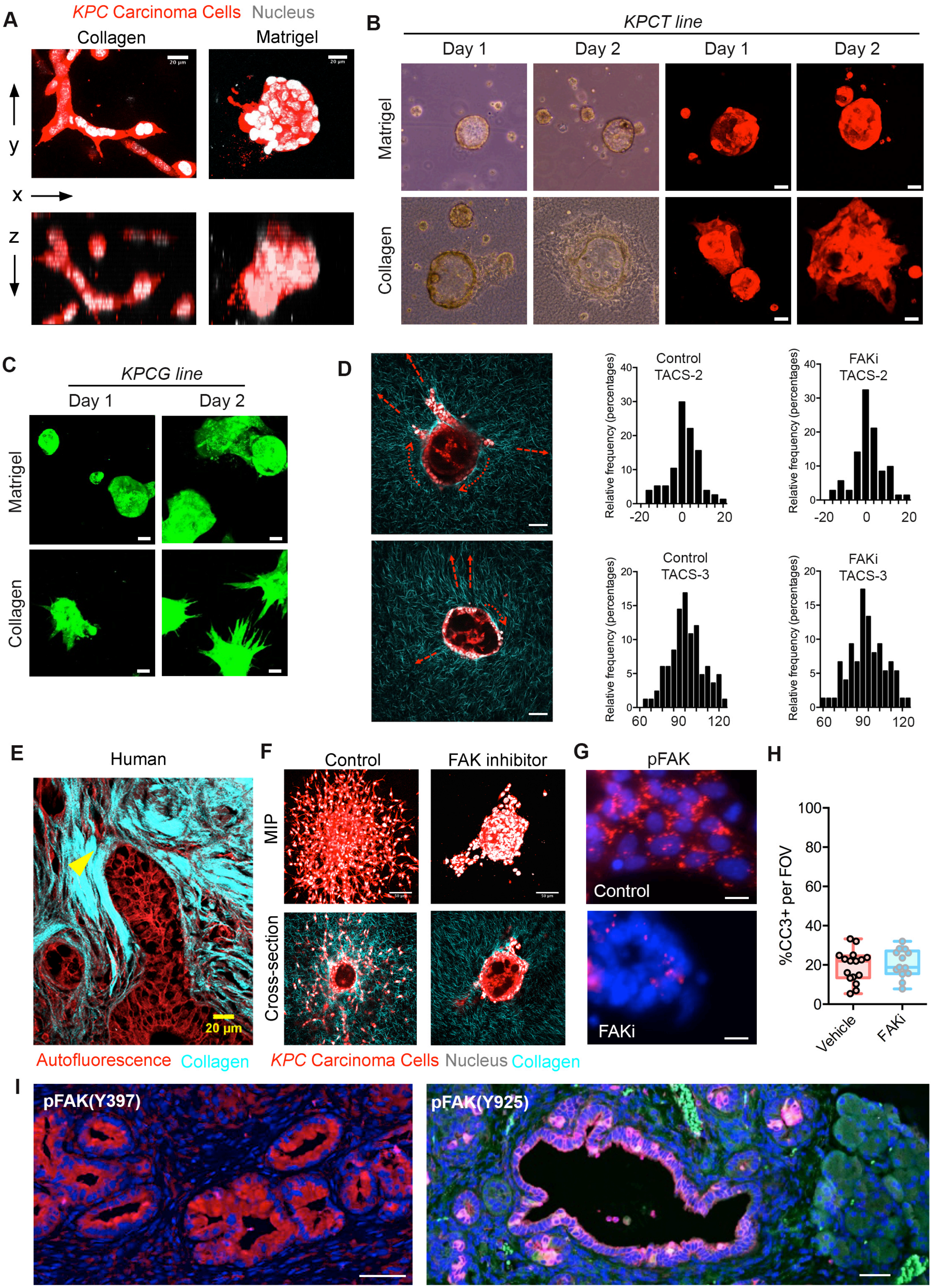
A
KPC Carcinoma Cells Nucleus
Collagen
Matrigel
y
x
z
B
KPCT line
Day 1
Day 2
Day 1
Day 2
Matrigel
Collagen
C
KPCG line
Day 1
Day 2
Matrigel
Collagen
D
Control TACS-2
FAKi TACS-2
Control TACS-3
FAKi TACS-3
Relative frequency (percentages)
E
Human
20 µm
Autofluorescence Collagen
F
Control
FAK inhibitor
MIP
Cross-section
KPC Carcinoma Cells Nucleus Collagen
G
pFAK
Control
FAKi
H
%CC3+ per FOV
Vehicle
FAKi
I
pFAK(Y397)
pFAK(Y925)

Supplementary Fig. 4: Single cell extrusion and dissemination *in vitro* and *in vivo*: (**A**) MPE micrographs of primary *KPCG* cells (pseudo-colored red) cultured in 3D collagen and 3D Matrigel showing contrasting morphologies with cells forming tight ductal-like clusters in Matrigel in contrast to more tubular, spindle-shaped, protrusive structures in 3D fibrous collagen matrices. (**B**) *KPCT* microtissues formed with microfluidic technology embedded in either Matrigel or collagen matrices showing that in predominantly basement membrane environments (i.e. Matrigel) the duct-like microtissues largely retain their epithelial phenotype while in collagen they begin to expand and invade. (**C**) Microtissues formed using a more invasive primary line (*from a KPCG mouse)* again shows a robustly invasive phenotype in collagen matrices while in Matrigel the microtissues retain a rounded non-invasive morphology. (**D**) Examples and quantification of TAC-2 (Curved lines) and TACS-3 (Straight lines) around embedded microtissues after 24 hours for both control and FAKi treated conditions. (**E)** A section from a human PDA sample shows collective invasion of the ductal boundary and subsequent single cell extrusion along TACS-3 (yellow arrowhead), akin to the group protrusions and single cell invasion observed in the epithelial organoids. (**F**) Combined MPE+SHG imaging presented as maximum intensity projection and cross-sectional views of cell extrusion and invasion from epithelial organoids into the surrounding collagen in Control and FAK inhibitor treated conditions at Day 3, demonstrating the drastic differences in extrusion efficiency. (**G**) Immunofluorescent staining for phosphorylated FAK (Y925; red) in carcinoma cells invading from microtissues and decreased pFAK following FAKi; blue = nuclear staining. (**H**) Evaluation of apoptosis from cleaved caspase-3 (CC3) immunofluorescence showing no differences between control conditions and FAK inhibitor (FAKi) treatment; n=13-16, n.s. by unpaired t-test. (**I**) Examples of phosphorylated FAK (Y397 in red: Left; Y925 in pink: Right) throughout PanIN lesions in pancreata from KPC mice; blue = nuclear staining. Scale bars= 10μm (G), 20 μm (A, E) and 50 μm (B, C, D, F, and I).

Movie Legends:

Movie 1: Surface rendering from a 3D multiphoton fluorescence micrograph of tdTomato expressing pancreatic carcinoma cells (*red*) from a live *KPCT* tumor explant showing extruded single cells from ductal structures.

Movie 2: Combined MPE and SHG imaging a live *KPCT* tumor explant showing fluorescent carcinoma cells (*red*) in ductal structures surrounded by collagen (*cyan*) undergoing coherent angular motion.

Movie 3: Combined MPE and imaging a live *KPCG* tumor explant showing two extruded carcinoma cells of small size and aspect ratio (*red*) interacting with periductal collagen (*cyan*) and switching phenotype.

Movie 4: Combined MPE and imaging a live *KPCT* tumor explant showing elongated single carcinoma cells (*red*) orienting and migrating along aligned periductal collagen tracks (*cyan*).

Movie 5: Combined MPE and imaging a live *KPCT* tumor explant simultaneously showing partially and fully extruded single carcinoma cells (*red*) associated with periductal collagen (*cyan*).

Movie 6: Combined MPE and SHG of *KPCT* microtissue embedded in a collagen matrix (*cyan*) at day 0 showing a stable epithelial morphology for carcinoma cells (*red*). Movie is imaging in 20min intervals over 12 hours.

Movie 7: Combined MPE and SHG of *KPCT* microtissue embedded in a collagen matrix (*cyan*) at day 3 showing extrusion and subsequent invasion of carcinoma cells (*red*). Movie is imaging in 20min intervals over 8 hours.

Movie 8: Combined MPE and SHG of *KPCT* microtissue embedded in a collagen matrix (*cyan*) under control conditions showing extrusion and subsequent invasion of carcinoma cells (*red*). Movie is imaging in 10min intervals over 10 hours.

Movie 9: Combined MPE and SHG of *KPCT* microtissue embedded in a collagen matrix (*cyan*) under FAK inhibition (FAKi) conditions showing inhibition of extrusion and invasion of carcinoma cells (*red*). Movie is imaging in 10min intervals over 10 hours.

Supplemental Methods:

Staining and imaging of archival tissues

FFPE sections were imaged on a custom-built multi-photon laser scanning microscope with 4-channel detection with interchangeable 440/20, 460/50, 525/50, 525/70, 595/50, 605/70, and 690/50 filters, (Prairie Technologies / Bruker) and a Mai Tai Ti:Sapphire laser (Spectra-Physics) to simultaneously produce MPE and SHG to visualize cells and collagen, respectively, which has been described in detail previously (1). To capture SHG from collagen, samples were imaged at 880-900nm and harmonic signal captured with a blue bandpass filter with a 440 or 450nm center wavelength. For human samples, the overall architecture of the tissue was visible by imaging endogenous fluorescence and was captured with a green emission filter at the same wavelength of excitation of 880-900nm. Alternately, some samples were stained with FITC-conjugated anti-pan Cytokeratin antibody (1:100, Sigma, F3418) to visualize carcinoma cells.

Since ZsGreen1 retained its fluorescence post-fixation, FFPE sections obtained from *KPCG* mice were imaged without additional staining for the carcinoma cells. ZsGreen1 fluorescence was excited by 880-900nm 2-photon excitation and detected through the green emission filter. Sections from *KPCT* mice were additionally stained with an anti-RFP antibody (1:100, Abcam, ab34771 and ab124754) and Alexa-conjugated secondary antibodies (Thermo Fisher) were used to visualize the tdTomato^{+} carcinoma cells post-fixation. The tdTomato fluorescence was imaged at 800nm two-photon excitation using the red emission filter sets (595/50). Note, all tdTomato or zsGreen fluorescence is pseudo-colored red in image throughout the manuscript for consistency of identifying carcinoma cells.

For staining FFPE sections (Cytokeratin or RFP), slides were first dewaxed and rehydrated in xylene and ethanol followed by antigen retrieval in Citrate buffer (pH 6.0) for 20mins in a steamer. Subsequently, the sections were blocked in 5% goat serum and incubated with the primary antibody overnight with 1% goat serum. The secondary was allowed to incubate for 40mins with 1:30000 bisbenzimide or Hoechst before washing and mounting with Prolong Gold. Alternatively, slides were subjected to antigen retrieval by steam heating in 1X Trilogy (Cell Marque). Slides were permeabilized with 0.025% Triton-X-100 (Roche) in 1X TBS (Bio-Rad) (TBS-T) and blocked for 1h at RT with 5% goat serum (Vector Labs) in 1X TBS-T. After blocking, for

immunofluorescence, slides were incubated overnight in a humidified chamber at 4 °C in TBS-T with 1% Fatty acid free-BSA (Fisher Scientific) with anti-mouse/human antibodies. The following primary antibodies were used: 1:50 mouse anti-Cytokeratin Pan-FITC (MilliporeSigma, F3418), 1:100 mouse anti-Pan-Keratin (Cell Signaling, 4523S), 1:200 rat anti-mouse CD31 (Dianova, DIA-310), 1:400 rabbit anti-CD31/PECAM-1 (Novus Biologicals, NB1002284), 1:400 rabbit anti-RFP (Abcam, ab34771 and ab124754), FAK(Y925) (1:100 Santa Cruz sc-11766) 1:200 rabbit anti-GFP (Life Technologies, A11122). Subsequently, slides were washed and incubated for 1h at RT with 1:200 Alexa-fluor secondary antibodies: goat anti-rabbit 488 (Life Technologies), goat anti-rat 568 (Life Technologies) along with 1:500 Draq5 (Biolegend) nuclear stain followed by wash steps and mounting with Prolong Gold (Life Technologies).

Engineering microtissues to analyze cell extrusion and invasion

Matrigel microtissues approximately 150-200μm in diameter were fabricated modifying previously established protocols (2, 3). Matrigel (Corning) was thawed on ice overnight and diluted to a concentration of 6 mg/mL with Dulbecco's phosphate-buffered saline (DPBS). At 4°C, the Matrigel solution was partitioned into droplets using a flow-focusing polydimethylsiloxane (PDMS) (Dow Corning) microfluidic device. The continuous phase from the droplet generation (FC-40 with 2% 008-FluoroSurfactant, Ran Biotechnologies), was collected with the droplets in a low retention Eppendorf tube and polymerized for 30 minutes at 37°C. The oil phase was removed and the Matrigel droplets were resuspended in 1x DPBS with a manual micropipette.

Droplets were then moved to agarose microwells, fabricated following previously established protocols (3). Briefly, polystyrene multi-well plates were coated with 2% agarose and dehydrated in a sterile laminar flow hood overnight. PDMS stamps with 300 μm diameter posts were plasma treated for 2 minutes to produce a hydrophilic surface and sterilized with boiling water. Agarose solution was pipetted into each well and the hydrophilic PDMS stamp placed immediately onto the molten agarose. After cooling for 5 minutes, stamps were removed gently from the polymerized agarose and hydrated with 1 x DPBS. Wells were washed with appropriate culturing media prior to adding microtissues with a manual micropipette.

Once inside the microwells, droplets were coated with primary *KPCT* or *KPCG* cells at a concentration of 100,000 cells/well. These microtissues were cultured in complete DMEM for 7 days to allow cells to adhere to the outside of the droplets and form luminal structures. Droplets were then treated daily with either 1μM FAK inhibitor VS-4718, or with vehicle control (DMSO) for three days, after which they were embedded in 4mg/mL 3D collagen-I gels (Corning), with continued inhibitor or DMSO treatment. Briefly, collagen gels were generated by mixing collagen-1 with an equal volume of 100mM HEPES buffer in 2X DPBS, as previously described (4-6). Droplets were added to this neutralized solution, which was then aliquoted out in 475uL increments into a 12-well culture plate and allowed to polymerize for 30 minutes at room temperature. Constructs were moved to 37°C for 4 hours before overlaying media. Embedded droplets were imaged using MPE and SHG imaging over days 0-4 days post-embedding. Invasion events were quantified by counting the number of cells protruding from luminal structures. Laminin staining was performed with 1:100 anti-laminin Ab (ThermoFisher MA106100) and 1:200 Alexa-fluor secondary antibody as described in the “Staining and imaging of archival tissues” section.

References:

1. Carlson M, Watson AL, Anderson L, Largaespada DA, and Provenzano PP. Multiphoton fluorescence lifetime imaging of chemotherapy distribution in solid tumors. *J Biomed Opt.* 2017;22(11):1-9.
2. Brett ME, Crampton AL, and Wood DK. Rapid generation of collagen-based microtissues to study cell-matrix interactions. *Technology.* 2016;4(2):80-7.
3. Crampton AL, Cummins KA, and Wood DK. A High-Throughput Workflow to Study Remodeling of Extracellular Matrix-Based Microtissues. *Tissue engineering Part C, Methods.* 2019;25(1):25-36.
4. Ray A, Slama ZM, Morford RK, Madden SA, and Provenzano PP. Enhanced Directional Migration of Cancer Stem Cells in 3D Aligned Collagen Matrices. *Biophys J.* 2017;112(5):1023-36.
5. Ray A, Morford RK, and Provenzano PP. Cancer Stem Cell Migration in Three-Dimensional Aligned Collagen Matrices. *Current protocols in stem cell biology.* 2018:e57.
6. Ray A, Morford RK, Ghaderi N, Odde DJ, and Provenzano PP. Dynamics of 3D carcinoma cell invasion into aligned collagen. *Integr Biol (Camb).* 2018;10(2):100-12.
